# The Melting Column as a Filter of Mantle Trace‐Element Heterogeneity

**DOI:** 10.1029/2018GC007880

**Published:** 2018-12-28

**Authors:** Tong Bo, Richard F. Katz, Oliver Shorttle, John F. Rudge

**Affiliations:** ^1^ Department of Mechanics and Engineering Science Peking University Beijing China; ^2^ Department of Earth Sciences University of Oxford Oxford UK; ^3^ Department of Earth Sciences University of Cambridge Cambridge UK; ^4^ Institute of Astronomy University of Cambridge Cambridge UK; ^5^ Bullard Laboratories, Department of Earth Sciences University of Cambridge Cambridge UK

**Keywords:** trace elements, mantle heterogeneity, melt transport, basalt, geochemistry, modeling

## Abstract

The observed variability of trace‐element concentration in basaltic lavas and melt inclusions carries information about heterogeneity in the mantle. The difficulty is to disentangle the contributions of source heterogeneity (i.e., spatial variability of mantle composition before melting) and process heterogeneity (i.e., spatial and temporal variability in melt transport). Here we investigate the end‐member hypothesis that variability arises due to source heterogeneity alone. We model the attenuation of trace‐element variability introduced into the bottom of a one‐dimensional, steady‐state melting column. Our results show that the melting column can be considered to be a filter that attenuates variability according to the wavelength of heterogeneity, the partition coefficient of the trace element, melt productivity, and the efficiency of melt segregation. We further show that while the model can be fit to the observations, this requires assumptions inconsistent with constraints on the timescales of magma assembly. Hence, we falsify the end‐member hypothesis and, instead, conclude that observed variability requires heterogeneity of melt transport. This might take the form of channels or waves and would almost certainly interact with source heterogeneity.

## Key Points

- Basalt trace‐element variability contains information about source and process heterogeneity
- A model of source heterogeneity shows that the melting column acts as a wavelength‐dependent filter of heterogeneity
- Source heterogeneity is not solely responsible for observed variability; process heterogeneity (channelized melt transport) must contribute

## 1 Introduction

Basaltic lava compositions can potentially constrain models of melting, melt transport, and the chemical character of the source mantle. Increasing attention has focused on the meaning of chemical variability at different length scales (e.g., Gurnis, 1986; Laubier et al., 2012; Neave et al., 2018; Shorttle, 2015). Some of this variability is inherited from mantle‐derived magmas that are the product of melting a heterogeneous source. The melting process and melt transport determine how that source is sampled by the segregating magma. Therefore, observed geochemical variability should contain a signal that represents a (conceptual) convolution of source and process. Deconvolving these requires a quantitative understanding of the factors that determine magma composition.

Two end‐members of such models could be imagined. In the first, the mantle source, prior to any melting, is homogeneous. Physical instability leads to spatio‐temporal variability of melt transport. The most prominent example is of channelized magmatic flow, arising by a reactive instability (e.g., Aharonov et al., 1995; Kelemen et al., 1995; Liang et al., 2010; Rees Jones & Katz, 2018; Spiegelman et al., 2001). Channels are hypothesized to transport deep, low‐degree, enriched melts to the surface without aggregating the depleted melts that are produced at shallower depths (Spiegelman & Kelemen, 2003). Magmatic solitary waves may also be capable of transporting deep, enriched melts in isolation from those produced shallower (Jordan et al., 2018).

The other end‐member is a heterogeneous mantle source with uniform melt transport. In this case, it is sufficient to consider a model domain that is one‐dimensional, aligned with the vertical. This end‐member can address only trace‐element or isotope heterogeneity, as these do not modify the melting rate (as would major‐element heterogeneity). Lithophile trace elements have low concentrations in the source, typically slow diffusivities in solid phases and distinct incompatibilities; they provide a useful indicator of magmatic processes. Large variations in the concentration of incompatible lithophile trace elements are routinely observed in suites of primitive basalts and melt inclusions. Isotopic evidence requires that some of this variation must be inherited from the mantle source (e.g., Maclennan, 2008b; Saal et al., 1998; Stracke et al., 2003). The model developed below addresses this inheritance in the context of laterally uniform melt transport. In particular, it considers the preservation or attenuation of trace‐element heterogeneity during simple, vertical melt transport, and aggregation.

### 1.1 Model Overview

In this paper we aim to understand the end‐member scenario of source‐heterogeneity with laterally uniform melt transport. We ask the questions: (i) Which wavelengths of heterogeneity are preserved in the magma during its segregation and ascent through an upwelling, one‐dimensional column of mantle rock? (ii) Which wavelengths of heterogeneity are filtered out? (iii) How does a trace element's partition coefficient affect the transfer of the source heterogeneity through the melting region? (iv) How does the low‐productivity tail of deep, low‐degree melting affect the transfer from source? The model we develop to answer these questions envisions one‐dimensional, vertical, steady melt segregation to the base of the crust.

Following DePaolo (1996), we regard mantle trace‐element heterogeneity as the sum of sinusoidal variations of different wavelengths. The peak and trough of the sinusoidal cycle reflect a source that is incompatible‐element *enriched* and *depleted*, respectively. It is important to note, therefore, that the model does not have a binary distinction between sources. Instead, the source composition is continuous and smoothly varying. We note that any smooth and periodic function can be constructed by an appropriately weighted sum of sinusoidal (Fourier) modes.

The chemistry described by the model is the partitioning and segregation of fictive trace elements. We define trace‐element equilibrium in the common way through partition coefficients, *K*, such that in equilibrium, *c* ^*s*^=*K* *c* ^*ℓ*^. The mass of trace elements is conserved. Conservation statements alone, however, do not constrain the way that elements are transferred between phases. A common description of melting in the geochemical literature is as a near‐fractional process (e.g., Asimow & Longhi, 2004; Beattie, 1993; Hellebrand et al., 2001; Johnson et al., 1990; McKenzie & O'Nions, 1991; Yoder, 1976), whereby incremental melts are in equilibrium with the composition of their parental solid prior to their near complete extraction. Here we adopt this approach and quantify its implications for the inheritance of chemical variability from the heterogeneous source.

Melting rate is an important model parameter, as it ultimately controls segregation of liquid from solid within the melting region. We model two idealized patterns of melting rate: *dry* mantle melting, in which the rate is constant from the solidus intersection to the base of the crust, and *wet* mantle melting, where a low‐productivity tail precedes an interval of nominally dry melting. Consideration of these two regimes is motivated by the expectation that the height of the partially molten region could be important. In particular, attenuation of source heterogeneity should be promoted if the melt region simultaneously contains multiple cycles of the source heterogeneity. Segregating melts of the chemically diverse sources aggregate and mix, which pulls the composition of enriched or depleted melts back toward the mean composition of the melting region. The more cycles of heterogeneity in the melting region, the greater is this regression to the mean. Dry and wet melting represent shorter and longer column lengths, respectively, and should thus behave differently.

In this context, reaction between melts and solid could also play a role in attenuating source heterogeneity. To explore this possibility, we assume that the aggregated melt can react with the solid to move toward chemical equilibrium. The reaction rate at which this occurs is proportional to the chemical deviation from equilibrium, as defined by the partition coefficient *K*. If reaction is infinitely fast, the model describes batch melting and equilibrium transport. For finite values of the reaction rate, considered in section 3.4, partial equilibration occurs. For zero reaction rate, the model describes fractional melting and disequilibrium transport; this combination is commonly assumed in geochemical models and is the focus of this paper.

The final consideration for the model is how to describe the output melt chemistry in relation to that of the source that is input at the bottom of the melting region. We quantify the transfer of incoming source heterogeneity to outgoing magma variability in terms of the admittance $A^{\ell }$, a concept developed further below. In short, it is analogous to the mean‐normalized variance for trace elements from a suite of basaltic lavas or melt inclusions.

### 1.2 Previous Melt Models Investigating the Transport of Source Heterogeneity

Many previously published studies employ column models that assume porous magmatic ascent, with full or partial aggregation of the melts produced at different depths. This is the basis on which McKenzie (1985, 1985) and Navon and Stolper (1987) developed theories for trace‐element transport, showing that equilibration between liquid and solid phases leads to transport rates that depend on the partition coefficient. Near equilibrium, heterogeneities of incompatible trace elements move at the chromatographic velocity, which is intermediate between the liquid and solid velocities and depends on the partition coefficient and melt fraction. Under idealized conditions (i.e., neglecting diffusion and dispersion), transport in equilibrium preserves chemical heterogeneities at all wavelengths. Kenyon (1990) and DePaolo (1996) found that dispersion causes attenuation at very short wavelengths of heterogeneity.

Navon and Stolper (1987) recognized that a long diffusion time is potentially required to equilibrate the melt with the interior of solid grains and that this will lead to a more substantial deviation from ideal, chromatographic behavior. Disequilibrium models that explicitly track diffusion along the radii of representative, spherical grains were developed to address this issue (Iwamori, 1992; 1993; Qin, 1992). They show that the effective partition coefficient can be significantly higher than the equilibrium value if transfer into the melt is rate‐limited by diffusion through the solid. Thus, partial equilibration essentially traps incompatible elements in the grain interior (see also Liang & Liu, 2016). However, all of these studies focused on steady‐state solutions, which precludes a treatment of chemical heterogeneity of the source.

Kenyon (1990) and subsequent papers (Kenyon, 1993; 1998) considered how disequilibrium transport could attenuate (or preserve) fluctuations of trace‐element concentration in ascending magma. Her models idealize pores as narrow, vertical sheets of magma that are interleaved with slabs of solid. Both magma sheets and solid slabs have uniform width; melt ascent rate is constant. Chemical equilibrium is imposed at the liquid‐solid interface. The liquid is assumed well mixed in the across‐pore direction with zero diffusion parallel to the flow; transport through the solid is by horizontal diffusion only. A sinusoidally varying concentration of trace elements, representing melt derived from a heterogeneous source, is injected into the bottom of the domain and modified by exchange with the solid. There is no melting in the interior of the domain.

Kenyon (1990) presents an analytical solution to this problem. The solution is discussed in terms of vertical transport rates and attenuation of heterogeneity amplitude. Both are considered as a function of oscillation frequency and pore width and spacing. The solid diffusivity is held at 10^−17^ m^2^/sec. Attenuation increases with frequency, such that melt oscillations with periods of order 1,000 years are eliminated over less than a kilometer of rise. For mantle upwelling at 3 cm/year, this period corresponds to a source wavelength of about 30 m. At the same upwelling rate, source heterogeneity wavelengths of order 10 km give periods of 10^5^ years. In Kenyon's models, these longer‐period oscillations attenuate over tens to hundreds of kilometers of rise.

Key to the question of disequilibrium during melt transport is knowing the trace‐element diffusivities. Rare‐Earth element diffusivities were measured by Van Orman et al. (2001) and found, in general, to be significantly smaller than assumed by Kenyon (1990). This would reduce the rate of melt equilibration with the solid and hence also reduce the attenuation of heterogeneity amplitude. Kenyon's theory would then predict preservation of shorter‐period oscillations. However, Kenyon's (1990, 1993, 1998) model neglects melting. It is well known that melting transfers trace‐element mass to the liquid phase over a finite range of melt fractions (which for decompression melting translates to a depth interval). This should logically play a role in the attenuation of heterogeneity.

Melting is included by Liu and Liang (2017) in a model of vertical, disequilibrium transport of trace‐element heterogeneities. Their analysis focuses on the stretching of isolated, noninteracting trace‐element anomalies. The use of isolated heterogeneities makes it difficult to generalize to a multiscale view of mantle heterogeneity. Liu and Liang (2017) concluded that smaller heterogeneities are more easily attenuated during melt segregation. This is reinforced by a more detailed paper by Liang and Liu (2018) as well as by the results presented below.

Here we focus on the transfer of heterogeneity from the mantle to the magma by progressive melting. We show that attenuation dominantly occurs by melt segregation during the initial (deepest) phase of melting. Our model assumes equilibrium melting and melt transport without chemical equilibration between melt and solid. Our key finding is that melt transport attenuates chemical heterogeneity of the upwelling mantle, depending on partitioning of the element considered, its lengthscale of variation in the source mantle, and the vertical structure of melting rate. This remains true for partial chemical equilibration. In melts delivered to the crust, wavelengths of order 1 km can be preserved only for the most incompatible elements.

### 1.3 Outline of Manuscript

The manuscript is arranged as follows. In section 2 we explain the domain, boundary conditions, and governing equations of the column model. In section 3 we illustrate the behavior of the model for simple scenarios of dry melting (with constant productivity) and wet melting (which adds a low‐productivity tail). We develop a physical argument for attenuation of trace‐element heterogeneity. And we examine the consequences of reactive equilibration of liquid and solid. Section 4 compares three observational data sets from the literature with model predictions in terms of the variance of concentration. Finally, section 5 discusses the model and its limitations. We return to the question of whether observed variability is a consequence of source heterogeneity or nonuniform melt transport. We conclude that source heterogeneity cannot fully explain the chemical diversity of basalts and that variability of melt transport (e.g., channelized flow) is required.

## 2 Model of Trace‐Element Transport

We consider a one‐dimensional domain aligned with gravity—a melting column. The top of the column is located at *z* = 0 and represents the Moho; the bottom of the column is located at *z* = *z*_0_<0, where |*z*_0_| is the depth at which upwelling mantle begins to melt and its porosity becomes nonzero.

The boundary condition at the bottom of the column represents the mantle composition as it upwells steadily into the domain at a rate *W*_0_. It has a mean, which is independent of time, and a sinusoidal fluctuation that represents the introduction of source heterogeneity. We can express this in terms of the complex expression

(1) $$c_{0}^{s}(t)={\bar{c}}_{0}^{s}+{\overset{˘}{c}}_{0}^{s}e^{i\Omega_{0}t},$$

where ${\bar{c}}^{s}$ is the steady part of the mantle concentration and ${\overset{˘}{c}}^{s}$ is the complex amplitude of the fluctuating part, and hence also determines the phase‐angle (recall Euler's formula, $e^{i\Omega_{0}t}=\cos\Omega_{0}t+i\sin\Omega_{0}t$). The subscript 0 indicates quantities at the bottom of the column.

The frequency of the fluctuating part of the boundary is

(2) $$\Omega_{0}=\frac{2\pi W_{0}}{\lambda_{0}},$$

where *W*_0_>0 is the mantle upwelling speed at the bottom of the column and *λ*_0_ is a wavelength of heterogeneity in the mantle prior to the onset of melting.

### 2.1 Governing Equations of Trace‐Element Transport

Conservation of mass equations governing trace‐element evolution in the solid (mantle, *s*) and liquid (magma, *ℓ*) phases are

(3a) $$(1-\phi )\rho \frac{D_{s}c^{s}}{Dt}=-\left(c^{\Gamma }-c^{s}\right)\Gamma -X,$$

(3b) $$\;\phi \rho \frac{D_{\ell }c^{\ell }}{Dt}=+\left(c^{\Gamma }-c^{\ell }\right)\Gamma +X,$$

where D_*j*_/D*t* is a Lagrangian derivative following a parcel of phase *j* (*s* or *ℓ*), *c* ^Γ^ is the trace element concentration in the instantaneously produced melt with infinitesimal mass per unit volume Γd*t*, and X is the rate of an interphase mass‐exchange reaction. Γ represents the melting rate (kg/m^3^/s); it is strictly positive in the models we consider, but we defer any further specification until later in the manuscript. Equations (3) state that the rate of change of trace‐element concentration in a moving parcel of solid mantle 3a or liquid magma 3b is due to interphase transfer by melting and by reactive exchange. Macroscopic diffusion and dispersion of trace elements are neglected for both phases.

Fractional melting and linear kinetics are specified by

(4) $$\;c^{\Gamma }=c^{s}/K,$$

(5) $$\;\;X=R\left(c^{s}-Kc^{\ell }\right),$$

where $K\equiv {\left[c^{s}/c^{\ell }\right]}^{\text{eq}}$ is a partition coefficient representing the equilibrium ratio of solid to liquid concentration and R is a kinetic coefficient with units kg/m^3^/s. Equation (4) states that the instantaneously produced melt is in equilibrium with the entire solid residue (there is no freezing in the model domain). Equation (5) states that the exchange of trace‐element mass between phases occurs at a rate that is linearly proportional to the difference from equilibrium. We take both *K* and R to be constant and uniform within any solution of equations (3) but explore their parametric control using suites of solutions.

For R→∞, reaction eliminates even the smallest deviations from trace‐element equilibrium, and hence, the column produces batch melts. In contrast, for R→0, reaction makes no contribution; fractional melts travel up the column but do not equilibrate with the residue they traverse. In this case, the column produces aggregated fractional melts. Below we explore the model behavior across this range and determine how large or small R must be to effectively obtain these end‐member regimes.

### 2.2 Expansion Into Trace Element Means and Fluctuations

The full solution to the problem can be expanded into steady and fluctuating parts (Liang, 2008). The steady part represents the mean concentration as a function of depth for all time; the fluctuating part represents the temporal oscillations associated with heterogeneity. The expansion is written:

(6a) $$c^{s}(z,t)={\bar{c}}^{s}(z)+{\overset{˘}{c}}^{s}(z)e^{i\Omega t},$$

(6b) $$c^{\ell }(z,t)={\bar{c}}^{\ell }(z)+{\overset{˘}{c}}^{\ell }(z)e^{i\Omega t},$$

where the functions ${\overset{˘}{c}}^{s}(z)$ and ${\overset{˘}{c}}^{\ell }(z)$ are the complex amplitudes of fluctuation, which depend only on depth. It is important to note that while mean concentrations must obey ${\bar{c}}^{s},{\bar{c}}^{\ell }>0$, the fluctuations must oscillate about zero so as to have zero time‐mean. The amplitude of the fluctuations is small enough that the full solid and liquid concentrations *c* ^*s*^,*c* ^*ℓ*^ are always positive. Only the real part of concentrations *c* ^*s*^ and *c* ^*ℓ*^ are physically relevant.

The time‐dependence in (6) has been expressed in terms of an oscillatory function with the same frequency for the liquid and the solid. The assumption of this form stems from the linearity of the equations; the frequency of the solution is locked to the frequency of the forcing at the boundary, equation (1). Therefore, Ω=Ω_0_ uniformly and for both phases.

Moreover, because the governing equations (3) are linear, superposition applies, and we can solve for the mean and fluctuations separately. Substituting (4), (5), and (6) into (3) and requiring the mean terms to balance give

(7a) $$(1-\phi )\mathrm{\rho W}\frac{d{\bar{c}}^{s}}{dz}=-\left({\bar{c}}^{s}/K-{\bar{c}}^{s}\right)\Gamma -R\left({\bar{c}}^{s}-K{\bar{c}}^{\ell }\right),$$

(7b) $$\;\;\;\;\mathrm{\phi \rho w}\frac{d{\bar{c}}^{\ell }}{dz}=+\left({\bar{c}}^{s}/K-{\bar{c}}^{\ell }\right)\Gamma +R\left({\bar{c}}^{s}-K{\bar{c}}^{\ell }\right).$$

At the bottom of the column, the mean concentrations satisfy ${\bar{c}}^{s}(z=z_{0})={\bar{c}}_{0}^{s}$ and ${\bar{c}}^{\ell }(z=z_{0})={\bar{c}}_{0}^{s}/K$. The system (7) is a set of coupled, linear, ordinary differential equations.

The equations for the fluctuating part of the solution are partial differential equations, but they can be converted to complex ordinary differential equations (ODEs) by applying the time derivatives in (3) to the expansion in (6). This gives

(8a) $$(1-\phi )\rho \left(i\Omega {\overset{˘}{c}}^{s}+W\frac{d{\overset{˘}{c}}^{s}}{dz}\right)=-\left({\overset{˘}{c}}^{s}/K-{\overset{˘}{c}}^{s}\right)\Gamma -R\left({\overset{˘}{c}}^{s}-K{\overset{˘}{c}}^{\ell }\right),$$

(8b) $$\;\phi \rho \left(i\Omega {\overset{˘}{c}}^{\ell }+w\frac{d{\overset{˘}{c}}^{\ell }}{dz}\right)=+\left({\overset{˘}{c}}^{s}/K-{\overset{˘}{c}}^{\ell }\right)\Gamma +R\left({\overset{˘}{c}}^{s}-K{\overset{˘}{c}}^{\ell }\right).$$

At the bottom of the column, the fluctuation amplitudes satisfy the fluctuating part of the boundary condition (1). In particular, ${\overset{˘}{c}}^{s}(z_{0})={\overset{˘}{c}}_{0}^{s}$ and ${\overset{˘}{c}}^{\ell }(z_{0})={\overset{˘}{c}}_{0}^{s}/K$.

The variable that is most relevant for comparison with observations is $\left|{\overset{˘}{c}}^{\ell }(0)\right|$, the amplitude of fluctuation in the liquid at *z* = 0, the top of the melting column. For any regime, this will be linearly proportional to the amplitude of forcing, $\left|{\overset{˘}{c}}^{s}(z_{0})\right|$. Hence, we define and study a pair of quantities called *admittance* (sometimes called the modulus of transfer),

(9) $$A^{s}\equiv \frac{\left|{\overset{˘}{c}}^{s}(z)\right|}{\left|{\overset{˘}{c}}^{s}(z_{0})\right|},\;\;A^{\ell }\equiv \frac{\left|{\overset{˘}{c}}^{\ell }(z)\right|}{\left|{\overset{˘}{c}}^{s}(z_{0})\right|}.$$

Admittance is a crucial concept in the analysis presented here. It represents the fraction of the column‐bottom heterogeneity that is present at some height in the column. In other words, it is the part of the signal that has not been attenuated at that height.

We will be particularly interested in the liquid admittance as a function of the heterogeneity wavelength *λ*_0_, given the parameters *K* and R. This is written as $A^{\ell }(\lambda_{0}|K,R)$, where the vertical line separates the independent variable, wavelength, from the problem parameters, partition coefficient, and reaction‐rate constant. Although the admittances are defined at any height *z* − *z*_0_ in the column, in this manuscript they will be evaluated and plotted at the top of the column (*z* = 0) unless otherwise specified.

## 3 Analysis of Melting Columns

Upwelling and melt production in the melting column is written in terms of equations for conservation of mass and momentum for two interpenetrating fluids, a liquid phase (the magma) and a creeping solid phase (the mantle; Mckenzie, 1984). Assuming that compaction stresses are negligible (Ribe, 1985; Spiegelman, 1993), the one‐dimensional expression of these equations can be written:

(10a) $$\phi +\phi_{0}\frac{w_{0}}{W_{0}}{\left(\frac{\phi }{\phi_{0}}\right)}^{n}\approx F\;\text{for}\phi \ll 1,$$

(10b) $$w=W_{0}\frac{F}{\phi },$$

(10c) $$\;\;\;W=W_{0}\frac{1-F}{1-\phi },$$

where *ϕ*,*F*,*w*, and *W* are all functions of *z*. This solution arises when permeability is related to porosity as *k*_*ϕ*_=*k*_0_(*ϕ*/*ϕ*_0_)^*n*^, where *k*_0_ is the permeability at reference porosity *ϕ*_0_ and *n* is a constant (e.g., von Bargen & Waff, 1986; Miller et al., 2014; Rudge, 2018). In 10a,

(11) $$w_{0}=\frac{k_{0}\Delta \mathrm{\rho g}}{\phi_{0}\mu }$$

is a characteristic, buoyancy‐driven melt speed for magma buoyancy Δ*ρ* *g* and viscosity *μ*. Uncertainty in the appropriate value of *k*_0_ for the mantle translates to uncertainty in the rate of melt segregation. Unless otherwise specified, we use *k*_0_=10^−12^ m^2^ and *n* = 2 in this paper. The degree of melting is denoted by *F*(*z*) and can be computed from a known melting rate Γ(*z*) as $F(z)=\int_{z_{0}}^{z}\Gamma (z)/\rho W_{0}\;dz$. Further details are provided in Appendix A.

We consider two simplified melting scenarios and their consequences for filtration of mantle heterogeneity. The first is a dry scenario, where melting begins at about 70‐km depth and proceeds with constant isentropic productivity to the surface. The second is a wet scenario, where melting begins at about 120‐km depth with the production of volatile‐rich melts at very low productivity; productivity then increases with ascent above 70 km. Both columns reach a total degree of melting of 23%.

In sections 3.1 and 3.2, below, we present results from the dry and wet scenarios. These are obtained by solving equations (7) and (8) with no reaction ( R=0), representing disequilibrium transport of aggregated fractional melts. The most important characteristics of the results are described and illustrated. All of these characteristics can be explained within a simple, unified theory, which is provided in section 3.3. With this theory for disequilibrium transport in place, we then revisit the dry and wet melting columns with partial equilibration ( R>0) in section 3.4.

### 3.1 Dry Column: Constant Melt Productivity

The model assumes a melting rate driven by decompression, with a uniform isentropic productivity Π≡*F*_max_/*z*_0_. The melting rate is then

(12) $$\Gamma =\rho W_{0}\Pi ,$$

and hence, the degree of melting, *F*(*z*)=Π(*z* − *z*_0_), is linear with height in the column. The resulting column model is illustrated in Appendix B for a case with *F*_max_=Π|*z*_0_|=0.23. See Appendices A and B for further details.

The solution *ϕ*(*z*), obtained analytically from equation 10a when *n* = 2, can be substituted into 10c and both of these into equation 8a for the fluctuations in the solid phase. Under disequilibrium melt transport ( R=0), this equation can be solved analytically (Appendix B) to give the solid admittance as

(13a) $$A^{s}={\left(1-F\right)}^{1/K-1}$$

(13b) $$\;\;\approx e^{-F/K}=e^{-(z-z_{0})/\lambda_{T}}.$$

The exact result 13a is identical to the well‐known fractional melting solution of 7a for the mean concentration in the residue (Shaw, 2006). The approximation 13b is valid for incompatible elements at small degrees of melting. It shows that the attenuation of fluctuations occurs over a melting interval F≲K. We refer to this interval as the *transfer regime* because it represents the region in which most of the trace element is transferred from the solid to the liquid. The height of the transfer regime *λ*_*T*_ becomes the characteristic lengthscale for the attenuation of chemical variability. For constant isentropic productivity Π,

(14) $$\lambda_{T}=K/\Pi .$$

The transfer regime will be important in understanding the admittance of trace elements in the liquid phase.

Equation 8b governing trace‐element fluctuations in the liquid phase does not have a fully general, analytical solution. However, we derive an analytical bound on the admittance

(15) $$A^{\ell }\leq {\bar{c}}^{\ell }/{\bar{c}}_{0}^{s}$$

in Appendix B. This inequality states that the admittance of the liquid phase can be no larger than the ratio of the mean liquid concentration to the mean source composition. In other words, for the liquid phase, heterogeneity is attenuated at least as fast as the mean concentration is diluted.

**Figure 1:**
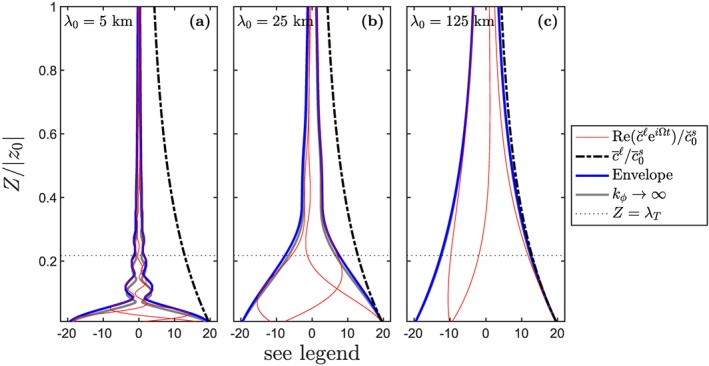
The vertical concentration structure of a trace element with K = 0.05 in a column of height |z_0_|=70 km, with uniform melt productivity and F_max_=0.23. The transfer regime height is λ_T_=D|z_0_|/F_max_≈15 km. Curves show the mean ${\bar{c}}^{\ell }$ (black) and fluctuations $\text{Re}\left({\overset{˘}{c}}^{\ell }e^{i\Omega t}\right)$ (red), normalized by the associated value in the unmelted mantle source (see legend for details). The wavelength λ of the input heterogeneity is (a) 5 km, (b) 25 km, and (c) 125 km. In each panel, three lines are plotted for $\text{Re}\left({\overset{˘}{c}}^{\ell }e^{i\Omega t}\right)$ evaluated at three different times by solving equation (8) numerically. Blue lines show the envelope for all possible times. Gray lines show the infinite‐permeability asymptotic model of equation (16). Details of the physical model for the melting column are given in Appendix B.

Numerical solutions to equation (8) are obtained using Runge‐Kutta methods. Figure 1 shows numerical solutions of trace‐element concentrations in the liquid as a function of height *Z* = *z* − *z*_0_ in the column. The fluctuations are plotted at three different times (red lines) by computing the real part of 6a, $\text{Re}\left[{\overset{˘}{c}}^{\ell }(z)e^{i\Omega t}\right]$. The envelope of the liquid fluctuations (blue lines) is given by the modulus of the fluctuation amplitude $\left|{\overset{˘}{c}}^{\ell }(z)\right|$. All of these curves represent an incompatible element with *K* = 0.05.

The three panels of Figure 1 show results for three wavelengths of heterogeneity, *λ*_0_=5, 25, and 125 km. Shorter wavelengths are more efficiently attenuated by the column than longer wavelengths. Indeed, the fluctuations of the *λ*_0_=5 km case (panel a) are qualitatively eliminated. Note that as predicted in equation (15), the envelope of fluctuations remains within the bound defined by the mean concentration. As the wavelength $\lambda_{0}\to \infty$, the envelope converges to the mean concentration.

We can understand the envelope structure in Figure 1 through an asymptotic analysis of the governing equation 8b (see Appendix B for details). When the permeability is taken to be infinite, upwelling of the liquid is much faster than that of the solid. In this limit (and for *K*,*F* ≪ 1), an asymptotic admittance can be computed exactly

(16) $$A^{\ell }∼\frac{\sqrt{1+e^{-2Z/\lambda_{T}}-2e^{-Z/\lambda_{T}}\cos(2\mathrm{\pi Z}/\lambda_{0})}}{F\sqrt{1+{\left(2\pi \lambda_{T}/\lambda_{0}\right)}^{2}}}.$$

This function is plotted in Figure 1 as gray lines that closely match the envelope obtained numerically. The gross decay of amplitude is controlled by the denominator of (16); the envelope fluctuations are controlled by the numerator. We consider each of these in turn.

For sufficiently small partition coefficient *K* we have *Z* ≫ *λ*_*T*_ near the top of the column. In this case, the numerator of (16) is ∼1, and we have

(17) $$A^{\ell }∼\frac{1}{F\sqrt{1+{\left(2\pi \lambda_{T}/\lambda_{0}\right)}^{2}}}\;\text{for}Z\gg \lambda_{T}.$$

Recall that *λ*_0_ is the wavelength of mantle heterogeneity in the source mantle. This equation indicates that near the top of the column, there are two admittance regimes. The first regime has *λ*_0_≫*λ*_*T*_, and hence, $A^{\ell }∼F^{-1}$, independent of *K* and *λ*_0_. This behavior is achieved for highly incompatible elements and/or for large heterogeneity wavelength. All source heterogeneity is mirrored in the melt, and hence, this is an upper bound on the admittance over parameter space. The second regime has *λ*_*T*_≫*λ*_0_, and hence, $A^{\ell }∼\lambda_{0}\Pi /(2\mathrm{\pi FK})$. Admittance thus decreases with partition coefficient and increases with wavelength and melt productivity.

Further down in the column, where *Z*/*λ*_*T*_ is *O*(1), the numerator of (16) plays a role. Oscillations in the envelope occur at the source‐heterogeneity wavelength *λ*_0_, but their amplitude decays over the transfer‐regime lengthscale. In the limit of *Z*→0, we can approximate the exponential and cosine functions with Taylor series and simplify to leading order to give $A^{\ell }∼1/K$. Hence, we note that the asymptotic behavior of admittance is closely related to the canonical fractional melting model at the top ( ${\bar{c}}^{\ell }/{\bar{c}}_{0}^{s}∼F^{-1}$) and bottom ( ${\bar{c}}^{\ell }/{\bar{c}}_{0}^{s}∼K^{-1}$) of the column.

**Figure 2:**
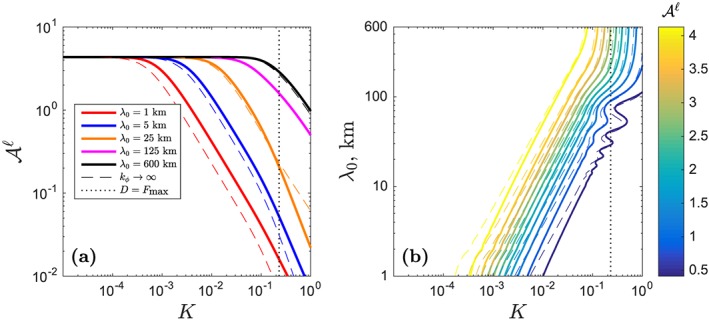
Admittance $A^{\ell }$ of trace‐element heterogeneity in the dry melting column (Figure 1) with maximum degree of melting F_max_ of 23%. Solid lines are obtained from numerical integration of equation 8b; dashed lines are calculated with the asymptotic solution (16). (a) $A^{\ell }$ as a function of partition coefficient K for various wavelengths of heterogeneity, as in legend. (b) Contours of constant $A^{\ell }$ as a function of K and input heterogeneity wavelength λ_0_. Other parameters as in Figure 1.

Figure 2 summarizes column‐model results for a range of heterogeneity wavelength and partition coefficient, in terms of the liquid admittance at the top of the column $A^{\ell }(z=0)$. The two panels are different ways of visualizing the same information: the filtration properties of the melting column. Panel (a) displays the two regimes that are identified by the infinite permeability model in equation (17). At small *K*, we are in the regime where *λ*_0_≫*λ*_*T*_ and hence where $A^{\ell }(0)∼F_{\max}^{-1}$. The column‐top admittance in this regime is independent of wavelength. At large *K*, we are in the other asymptotic regime that has $A^{\ell }(0)\propto \lambda_{0}/K$. Considering the full range of *K* in panel (a), we note that heterogeneity at a 1‐km wavelength is severely attenuated by transport through the column, except at the lowest partition coefficients (e.g., Barium, *K*≈10^−4^). In contrast, heterogeneity at a 125‐km wavelength is generally preserved in the column‐top aggregated melts.

Panel (b) of Figure 2 shows the same numerical results, plotted in terms of contours of equal $A^{\ell }$ in a wavelength–partition coefficient space. The thin, dashed lines are contours of the infinite‐permeability model (16), evaluated at the column top. In the upper‐left region of this plot, where both the column height and the heterogeneity wavelength are much greater than the transfer regime (*Z*_max_,*λ*_0_≫*λ*_*T*_), admittance is uniformly high ( $A^{\ell }∼F_{\max}^{-1}$), and heterogeneity is preserved. Moving from this region to the right takes us toward the regime where *λ*_*T*_≫*λ*_0_. To leading order, admittance in this regime varies as $A^{\ell }\propto \lambda_{0}/K$ (hence the contours have a slope ∼1).

In Figure 2b, the oscillations in admittance near *K* = *F*_max_ arise from the sinusoidal term in equation (16). The deviations from the overall trend are small, however, and occur only when the admittance is already low. Hence, the systematics of $A^{\ell }$ as a function of heterogeneity wavelength and partition coefficient is well‐described by equation (17). This equation rests on the assumptions of rapid melt segregation and a column that is much taller than the transfer regime. A more physically detailed explanation for the systematics of admittance is provided in section 3.3, below.

### 3.2 Wet Column: Variable Melt Productivity Due to Volatiles

**Figure B1:**
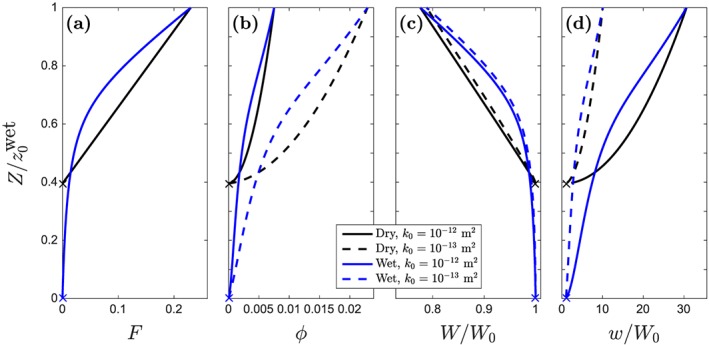
Melting column solutions for the two melting models considered here. Black lines show a prescribed, constant isentropic productivity (dry); blue lines show the two‐component model representing the effect of volatiles (wet). Two values of the reference permeability k_0_ are considered. (a) Degree of melting; (b) Porosity; (c) Solid upwelling rate; (d) Liquid upwelling rate. Parameters for all curves include permeability exponent n = 2; viscosity μ = 10 Pa s; density with the Boussinesq assumption ρ= 3,000 kg/m^3^; density difference between the solid and liquid phase Δρ = 500 kg/m^3^; F_max_=0.23, and W_0_=4 cm/year. The volatile here is water with a partition coefficient K_w_=0.01; source volatile concentration 100 ppm; heat capacity c_P_=1,200 J/K; Clausius‐Clapeyron slope γ = 5.54 × 10^−6^ Pa/K; specific latent heat L = 10^6^ J/kg; coefficient of thermal expansion α = 3×10^−5^ K^−1^; volatile solidus depression M =− 4 K/ppm; reference mantle temperature $T_{0}^{S}=1,600$ K.

We next consider a melting column model with a mantle source that contains volatiles (e.g., water and carbon). Although these volatiles are present in small concentration, they drastically lower the solidus temperature at a given pressure (e.g., Dasgupta & Hirschmann, 2006). Therefore, melting begins at a higher pressure. More importantly, the degree of melting *F* does not increase linearly with height in the column, as it did in the dry column model. The melting rate can still be described as in (12), but the productivity Π is no longer constant; it now depends on *z*, and so we replace it with d*F*/d*z*, which is a function of *z*. The zero‐compaction‐length column solution is given by (10), but with a nonlinear *F*(*z*). Details of this model are given in Appendix C (and Figure B1). In the present treatment, the volatile is taken to be water with a partition coefficient *K*_*w*_=0.01. Melting proceeds to the same final extent, however: *F*_max_=0.23. In Appendix C, a simple thermochemical model is introduced, where *F* is expressed as a function of temperature and *T*(*z*) is obtained by numerical solution of an energy conservation equation.

**Figure 3:**
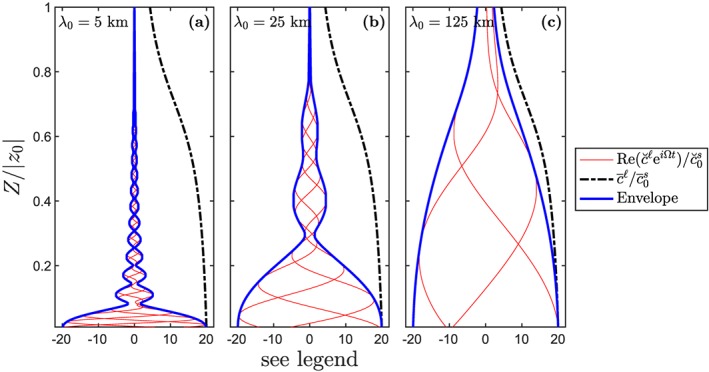
Melting column models with wet mantle source containing 100‐ppm water. Panels and lines as in Figure 1. The onset of wet melting is at 120 km depth and proceeds with nonuniform isentropic productivity. Details of the melting column physical and thermo‐chemical models are given in Appendix C and Figure B1.

Figure 3, as for Figure 1, displays solutions for ${\bar{c}}^{\ell }(z)$ and ${\overset{˘}{c}}^{\ell }(z)$ for a trace element with *K* = 0.05. The trace‐element concentrations in the liquid phase are plotted as a function of height *Z* = *z* − *z*_0_ in the column (with *z*_0_=−120 km). The mean (black line) is separated from the fluctuations (red lines), which have an envelope given by the blue lines. The fluctuating part is computed at three different times. From these curves we can draw a similar conclusion as in section 3.1. Shorter‐wavelength fluctuations are more efficiently filtered by the melting column than longer wavelengths. The envelope of fluctuations remains within the mean concentration, in agreement with the analytically derived bound in equation (15), which was obtained for the dry model. Moreover, as the wavelength $\lambda_{0}\to \infty$, the envelope converges to the mean concentration.

The wet column model has an onset of melting that is much deeper: 120 km versus 70 km for the dry case. It also has a nonconstant productivity of isentropic decompression d*F*/d*z*; indeed, there is a low‐productivity *tail* at depths below about 60 km. The depth axis is normalized by the column height in Figure 3, so a direct comparison to depths in Figure 1 is not straightforward. But it is clear that the black curves showing the canonical fractional melting solution differ between the wet and dry columns. A larger height‐fraction of the wet column has low *F* and hence high ${\bar{c}}^{\ell }/{\bar{c}}_{0}^{s}$. The envelope for the fluctuating part of the trace‐element concentration (blue curve), however, diverges from its upper bound deeper in the wet column than in the dry column—both in the relative terms of the fractional height and in the absolute depth.

**Figure 4:**
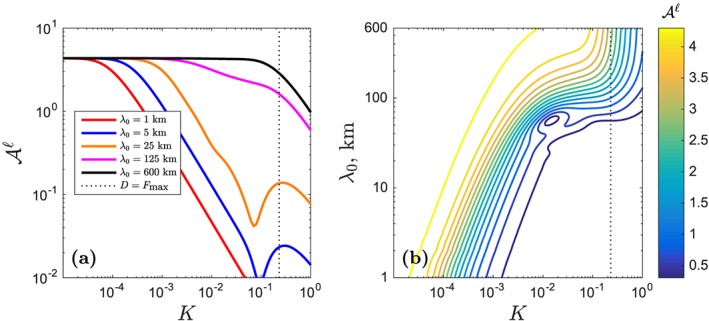
Admittance $A^{\ell }$ of trace‐element heterogeneity in the wet melting column with maximum degree of melting F_max_ of 23%. Panels and parameters as in Figure 2 except for the source water content of 100 ppm.

Figure 4, as in Figure 2, summarizes the behavior of the admittance for a suite of wet column model calculations. $A^{\ell }$ is plotted as a function of mantle heterogeneity wavelength *λ*_0_ and partition coefficient *K*. The general trend for the wet columns is the same as for the dry model: Heterogeneity is transported to the surface with less loss of amplitude when *K* is small and when *λ*_0_ is large.

However, comparing Figures 4 and 2 in more detail, there are significant differences in the admittance structure. Lines in Figure 4a show a more pronounced drop‐off when compared with Figure 2 (except the black line), and correspondingly, in Figure 4b, the contours shift leftward. Both panels indicate that the liquid admittance $A^{\ell }$ becomes smaller with the existence of volatiles. In other words, volatiles enhance the attenuation of mantle heterogeneity.

**Figure 5:**
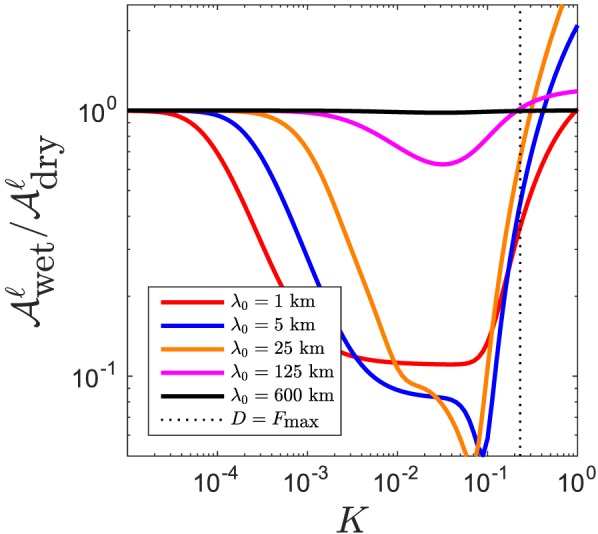
Ratio of admittance in the volatile model $A_{\text{wet}}^{\ell }$ to admittance in the simple model $A_{\text{dry}}^{\ell }$ as a function of partition coefficient K for various wavelengths of heterogeneity (see legend). Parameters are the same as those in Figures 2 and 4.

This enhanced attenuation is certainly evident when *K* ≪ *F*_max_. However, for partition coefficients that approach *F*_max_, nonmonotonic behavior appears in the curves of $A^{\ell }$ (Figure 4a). In Figure 5, a plot of the ratio of admittance in the wet and dry cases $A_{\text{wet}}^{\ell }/A_{\text{dry}}^{\ell }$ highlights this behavior. Where the wet/dry admittance ratio is less than unity, the wet column is more attenuating than the dry column. The ratio increases toward unity as *K*→*F*_max_ from below and, for some wavelengths, even exceeds unity. The black line, for a wavelength of heterogeneity of *λ*_0_=600 km, shows that at sufficiently long wavelength, the filtration effects of wet and dry columns are indistinguishable.

There are other irregularities of the curves in Figures 4 and 5. These generally occur when $A^{\ell }$ is already significantly less than unity, so they are of no practical importance and are not discussed further.

Above we have described results for trace element transport and filtration of heterogeneity signals in dry and wet melting columns. The most salient features have been highlighted, but no explanation was provided. In the next section, we explain all of these results within a single conceptual and quantitative framework. This framework may be usefully applied beyond the simple, one‐dimensional models presented here.

### 3.3 A Simplified Theory of Wavelength Selection

**Figure 6:**
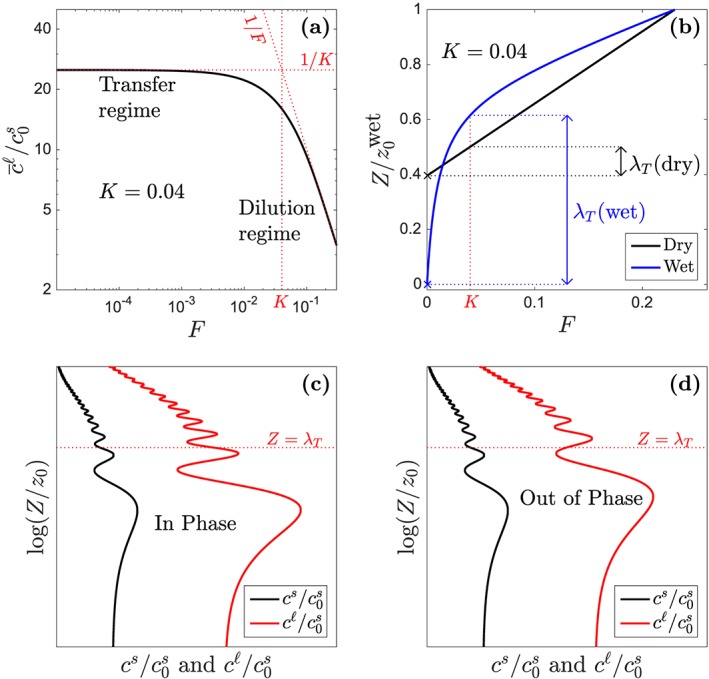
Plots to illustrate the mechanism of attenuation. (a) The canonical model of fractional melting (K = 0.04), plotted in a log‐log space. The red line at F = K delimits the transfer regime and the dilution regime. The liquid concentration approaches 1/K in the transfer regime, whereas it approaches 1/F in the dilution regime. (b) The length of transfer regime in the simple model and the volatile model. Black lines show the dry case; blue lines show the wet case. The red line denotes F = K with K = 0.04. Bottom panels are schematic diagrams showing how solid and liquid concentration can be (c) in phase or (d) out of phase in the transfer regime. Red lines represent the liquid phase; black lines represent the solid phase. Horizontal dotted lines mark Z = λ_T_, the upper boundary of the transfer regime, where F = K.

For any trace element with a fixed value of *K*, the vertical evolution of an aggregated fractional melt has two regimes: one at depths where *F*(*z*) < *K* and one where *F*(*z*) > *K*. Figure 6a shows that there is a significant change in trace‐element variation with *F* across this boundary. In the transfer regime, incremental melts transfer trace‐element mass from the solid to the liquid, keeping the liquid concentration nearly constant. In the dilution regime, the solid is depleted, and incremental melts only dilute the concentration of the liquid. These two regimes map onto the steady, one‐dimensional melting column because at any depth (and corresponding *F*), the mean liquid concentration is equal to the closed‐system, aggregated melt of the mean initial source concentration.

In a melting column, the transfer regime occurs toward the bottom, where *F*(*z*) < *K*, and the dilution regime holds toward the top, where *F*(*z*) > *K*. Trace‐element source heterogeneity is transferred into the liquid in the transfer regime and gets diluted in the dilution regime. For elements with *K* ≪ *F*_max_, dilution affects the admittance $A^{\ell }$ uniformly; this creates the upper bound on $A^{\ell }$ in Figures 2 and 4. Elements with $K≳F_{\max}$ are incompletely transferred to the liquid phase, and hence, their $A^{\ell }$ never reaches the upper bound of 1/*F*_max_.

However, at a fixed *K* ≪ *F*_max_, Figures 2 and 4 show that smaller wavelength of heterogeneity *λ*_0_ is associated with smaller $A^{\ell }$. This additional attenuation cannot take place in the dilution regime because melting of the depleted solid there dilutes trace elements independent of their wavelength of variation.

Attenuation of trace‐element variations in the liquid can occur in the transfer regime, where the solid retains a significant fraction of the total amount of trace element. Then the difference in the phase angle of oscillation between the liquid and solid causes the attenuation. If spatial variations in the liquid and solid remain *in phase*, then additional fractional melting increases the variability of the liquid; this is shown in Figure 6c. If the spatial variations go *out of phase*, as shown in panel (d), then fractional melting transfers higher‐than‐average concentrations where the aggregated melt has a lower‐than‐average concentration (and vice versa). This reduces variability in the liquid phase. Hence, it is phase differences within the transfer regime that cause attenuation of trace‐element variability and reduce $A^{\ell }$.

At the bottom of the melting column, where *F* = 0, the solid and liquid concentrations are in phase. Previously, we defined the height *λ*_*T*_ of the transfer regime as the interval of *z* over which *F* ranges from 0 to *K*. Figure 6b shows how *λ*_*T*_ is defined for dry and wet models for a given *K*. A phase shift arises within this height interval if the melt and solid travel at different speeds. Furthermore, if the wavelength of heterogeneity is small compared to *λ*_*T*_, then it is easier for a speed difference (i.e., for melt segregation) to cause a phase shift. The amount of attenuation, and hence the reduction in admittance, should scale with the average difference of phase‐angle between the liquid and the solid.

This can be clarified by considering the real part of the integrand in the expression for the liquid admittance (B7). Although the full equation is more complicated, its essence is evident in this term. It is also helpful to make the approximation ${\left(1-F\right)}^{1/K-1}\approx e^{-F/K}$ to give

(18) $$e^{-F/K}\cos\left[\Omega \left(t^{s}-t^{\ell }\right)\right].$$

This expression has two parts. The exponential part represents the mean transfer of concentration from the solid to the liquid; it highlights the characteristic melting scale over which the solid becomes depleted. The cosine term represents the effect of phase‐angle difference between the solid and liquid. In particular, *t* ^*s*^−*t* ^*ℓ*^≡Δ*t*(*F*) is the difference in transit time for the solid and the liquid to travel from the bottom of the melting column to the height *Z*, at which the degree of melting is *F*.

**Figure 7:**
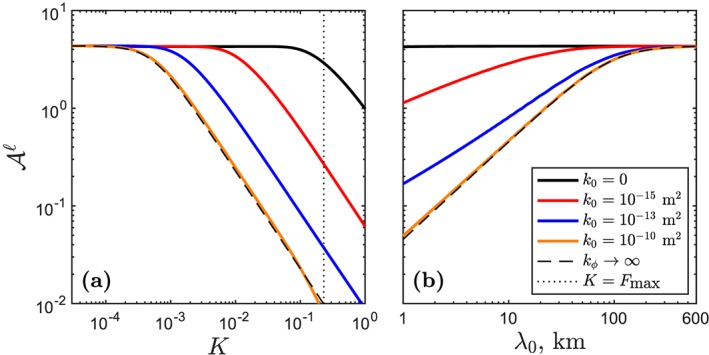
The control of permeability on admittance. Solid lines in both panels correspond to different values of reference permeability k_0_. Other parameters as in Figure 1. The asymptotic solution (dashed line) is computed with equation (17). (a) Admittance as a function of partition coefficient in a dry column for a heterogeneity wavelength λ_0_=1 km. (b) Admittance as a function of wavelength of heterogeneity for partition coefficient K = 0.05. The reference permeability used elsewhere in this paper is k_0_=10^−12^ m^2^.

Figure 7 shows how the rate of melt segregation controls the admittance. If the permeability approaches zero, solid and melt travel together, and there is no phase‐angle difference: Δ*t*(*F*) ∼ 0. In this case, attenuation of fluctuations is identical to dilution of the mean (this is the upper limit of the bound (15)). If, at the other extreme, the melt moves infinitely fast, then Δ*t*(*F*) ∼ *t* ^*s*^. In this case, the liquid aggregates instantaneous melts from the solid at all phase angles that fit between the bottom of the column and height *Z*(*F*). For finite values of permeability, between these two extremes, the admittance curves take intermediate values. As the reference permeability *k*_0_ becomes large, admittance curves in Figure 7 approach the lower‐bound asymptotic result for infinite permeability (17).

Panel (a) of Figure 7 plots admittance as a function of partition coefficient for *λ*_0_=1 km. Larger partition coefficients have a taller transfer regime, providing a longer *runway* for melt segregation, and hence generate phase‐angle differences that cause attenuation. Panel (b) plots admittance as a function of wavelength for *K* = 0.05. The height of the transfer regime is fixed, but as *λ*_0_ increases, the number of heterogeneity wavelengths that fit into the transfer regime decreases. This reduces the phase‐angle difference created by melt segregation.

Returning to the expression (18), we emphasize that the dominant contribution to the admittance is made when F≲K (when exp(−F/K) is of order unity). Hence, for highly incompatible elements (*K* ≪ *F*_max_), the ratio of wavelength to transfer‐regime height *λ*_0_/*λ*_*T*_ is the crucial control. This is expressed in equations (16) and (17), above. In summary, the expression (18) therefore tells us that heterogeneity wavelength, partition coefficient, adiabatic productivity, and the rate of melt segregation are all controls on the attenuation of trace‐element variability.

With this in mind, we return to the enhanced attenuation seen in wet melting column. There, the low‐productivity tail creates a larger *λ*_*T*_ at any given value of *K*, as shown in Figure 6b. Larger *λ*_*T*_ allows for more magma segregation within the transfer regime and thus greater Δ*t*(*K*) and more attenuation. The comparison between wet and dry admittance in Figure 5 shows that the ratio $A_{\text{wet}}^{\ell }/A_{\text{dry}}^{\ell }$ goes to 1 when *K* > *F*_max_. In this range of *K*, *λ*_*T*_ is equal to the full column height; the effect of increasing *λ*_*T*_ with a low‐productivity tail is negligible, especially since segregation is relatively slow at small porosity.

We can also now understand the waviness of attenuation contours in Figures 2b and 5b. These oscillations appear when the column height is similar to or greater than the height of the transfer regime (or, equivalently, when $F_{\max}≳D$). In these cases, the solid throughout the column retains some of the trace element and hence contributes to attenuation. Then the attenuation is higher (and $A^{\ell }$ lower) when an integer number of solid heterogeneity wavelengths fit into the column height. If an extra half‐wavelength fits, then $A^{\ell }$ is higher. For the infinite permeability model of equation (16), this is expressed by the cosine term in the numerator, taking *Z* = *Z*_max_ for the column top.

### 3.4 The Role of Exchange Reactions Toward Equilibrium

In this section, we consider the exchange of trace‐element mass between solid and liquid phases that drives the system toward equilibrium. This corresponds to the parameter regime with R>0. The reaction rate R is scaled by a reference melting rate, Γ_0_≡*ρ* *W*_0_Π≈1.25 × 10^−11^ kg/m^3^/s, where we used parameter values as in Figure B1.

**Figure 8:**
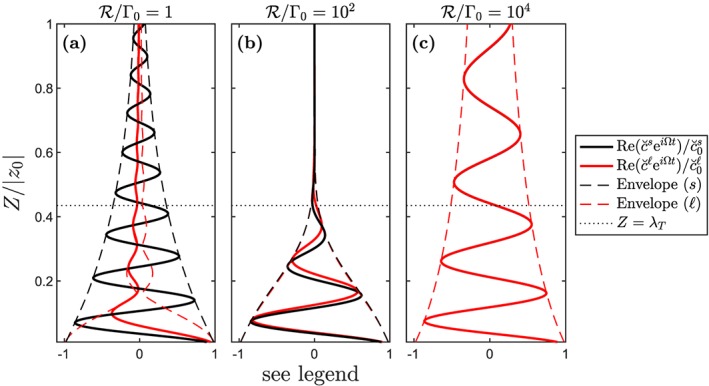
Vertical structure of fluctuations in the liquid and solid phase, $\text{Re}({\overset{˘}{c}}^{s}e^{i\Omega t_{0}})$ (black) and $\text{Re}({\overset{˘}{c}}^{\ell }e^{i\Omega t_{0}})$ (red), of a trace element with K = 0.1 and λ_0_=10 km, for three different reaction rates. Melting is computed assuming a dry column. Solid fluctuations are normalized by the initial value in the unmelted mantle source; liquid fluctuations are normalized by that in the incipient melt. The scaled reaction rate is (a) $R/\Gamma_{0}{=}^{1}$, (b) $R/\Gamma_{0}=10^{2}$, and (c) $R/\Gamma_{0}=10^{4}$. The solid lines are plotted for an arbitrarily chosen time t; dashed lines show the envelope of fluctuations.

Figure 8 shows trace‐element concentration in the liquid and solid for three values of R that span the behavioral spectrum. The column has dry melting with *K* = 0.1 and *λ*_0_=10 km. In panel (a), R=0 (as in the sections above), giving complete disequilibrium transport; the phase‐angle difference between the liquid and solid phases in the transfer regime controls the attenuation. In panel (c), the reaction rate is large enough that the trace element is in approximate equilibrium: ${\overset{˘}{c}}^{s}\approx K{\overset{˘}{c}}^{\ell }$ for all *Z*. The liquid and solid fluctuations remain in phase throughout the column and move upward with the chromatographic velocity (Navon & Stolper, 1987). Attenuation in this quasi‐equilibrium case is independent of *λ*_0_; instead, it depends only on *K*/*F*_max_. Indeed, below we demonstrate that admittance is generally maximized for R→∞.

Figure 8b shows the case of intermediate R, where exchange reactions move the system toward trace‐element equilibrium but are not fast enough to achieve it. The phase‐angle difference between the solid and liquid curves is nonzero. Attenuation of liquid fluctuations occurs by interphase transfer, but it also occurs by exchange reactions. This combination can lead to greater attenuation (and hence smaller $A^{\ell }$) than at either of the reaction‐rate extremes.

**Figure 9:**
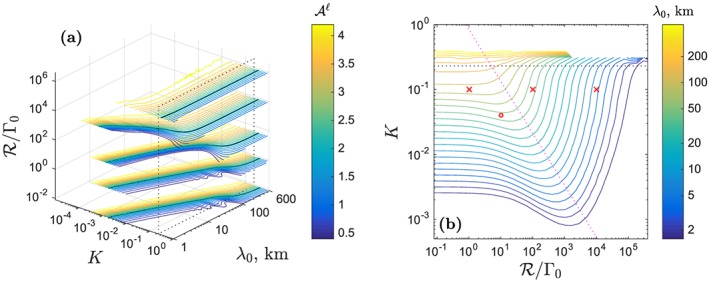
The systematics of liquid admittance as a function of partition coefficient, heterogeneity wavelength, and reaction rate for dry melting. (a) Contours of $A^{\ell }$ in the three‐dimensional space of K, λ_0_, $R/\Gamma_{0}$ at values of $R/\Gamma_{0}=8\times \{10^{-3},10^{-1},5,10^{3},10^{5}\}$. Black contour shows $A^{\ell }=2$. The set of contours at the smallest value of $R/\Gamma_{0}$ are almost identical to those in Figure 2b. (b) Contours of λ_0_ on a surface defined by $A^{\ell }=2$. Points marked with a red × are the conditions of the three panels in Figure 8; the red circle corresponds to $K=0.04,R/\Gamma_{0}=10$. The magenta dotted line has a slope of −1. In both panels, the black dotted lines indicate the position of K = F_max_.

Figure 9 shows the systematics of $A^{\ell }$ as a function of *K*, *λ*_0_, and R for dry melting‐column calculations. Panel (a) displays the full, three‐dimensional space with contours of $A^{\ell }$ plotted at five values of $R/\Gamma_{0}$. First we consider the set of contour lines at the smallest value of $R/\Gamma_{0}$. These are nearly identical to the contours in Figure 2b because reaction plays almost no role in equilibrating the solid and liquid. In this set of contours, at wavelengths $\lambda_{0}≳100$ km, the admittance becomes nearly independent of *λ*_0_ because there is almost no phase‐angle difference between the solid and the liquid concentration profiles. Hence, for very large wavelengths of heterogeneity, the system is in approximate equilibrium with respect to the partition coefficient despite melt segregation and the lack of reaction.

Moving to higher reaction rates, the quasi‐equilibrium regime extends toward smaller wavelengths. This is because reaction tends to eliminate any phase‐angle difference that would be created by segregation (cf. Figure 8c). For the fastest reaction rates considered, admittance becomes independent of wavelength for heterogeneities at scales greater than 1 km.

Another notable feature of Figure 9a is evident by comparison of all sets of contours at *K* = 0.1 and *λ*_0_=10 km (cf. Figure 8a and 8b). Under these conditions, admittance decreases with increasing reaction rate and then increases again. The former is due to reaction acting on (but not eliminating) differences in phase angle; the latter occurs as reaction drives the system into the equilibrium regime.

Figure 9b is a different view of the effect of reaction rate. Here we plot contours of the wavelength *λ*_0_ at which $A^{\ell }=2$. The contours indicate the smallest wavelength of heterogeneity that can be preserved under various conditions of reaction rate and partition coefficient. Following a horizontal line at, say, *K* = 10^−2^ from low to high R, wavelength increases slightly (more attenuation due to reaction) before decreasing sharply (less attenuation in the quasi‐equilibrium regime). The sharp change from the disequilibrium regime to the quasi‐equilibrium regime occurs across a boundary with a slope of −1 on this diagram.

Experimental measurements of trace element diffusivity indicate that it is extremely small (Van Orman et al., 2001). For example, for Neodymium in a spherical grain of radius *a* = 3 mm at a pressure of 1 GPa and temperature of 1,300 ° C, the reaction rate would be

(19) $$R∼\frac{4\pi \rho D^{s}}{a^{2}}\approx 1\times 10^{-10}{\text{kg/m}}^{3}/s,$$

where $D^{s}$ is the diffusivity in the solid. This estimate corresponds to $R/\Gamma_{0}\approx 10$. For a partition coefficient of *K*≈0.04, this sits in the disequilibrium regime (red circle in Figure 9b) but is rather close to the transition to chromatographic transport.

Cast in terms of a characteristic equilibration time, the above gives approximately one million years for Nd. At intermediate mid‐ocean ridge spreading rates, one million years is enough time for solid mantle to upwell through roughly half of the silicate melting regime beneath the axis. Hence, for ∼3‐mm grain size, we consider diffusive reequilibration of trace elements to be slow. But the quadratic dependence of R on grain size means that smaller grains will equilibrate much faster. There are few constraints on grain size in the asthenosphere, however, and models remain speculative.

Differences in diffusivity between trace elements may help to explain anomalies in their behavior, relative to a model based on equilibrium partitioning. These effects would be of second order, however, whereas the questions motivating this study pertain to observations of first‐order patterns.

## 4 Comparison With Observations

Model predictions can be compared with observations of trace‐element variability by making assumptions about the characteristics of heterogeneity that enters the bottom of the melting column. In particular, we must prescribe a time series of concentration for each trace element in the source mantle. This is largely unconstrained, and so we make simplifying assumptions. The key assumption is that the input heterogeneity is identical for all trace elements; that is, it is independent of *K*. The theoretical framework proposed here requires only that the time series be periodic; we can then analyze it in terms of its decomposition into Fourier modes. Below, after a discussion of the geochemical data sets, we formulate a synthetic representation of periodic heterogeneity that is suitable.

In section 4.1, we discuss the synthetic heterogeneity signal and describe models that aim to fit observational data. We use only dry column models but consider mantle heterogeneity with different periodicity, for comparison with observations. Then, in section 4.2, we summarize published geochemical observations from eruptions in Iceland and from a set of mid‐ocean ridge basalts (MORBs) sampled from the Central Indian Ridge. The data are considered in terms of their variance for each measured trace element. Importantly, the data sets all show a roughly log‐normal distribution of concentrations for each element. This motivates a hypothesis for the form of a synthetic heterogeneity.

### 4.1 Synthetic Heterogeneity

Constructing model instances to compare with observations involves specifying the parameters of the melting column (e.g., *z*_0_ and *F*_max_) as well as the details of the input heterogeneity. Thus far, we have considered only heterogeneity patterns consisting of sinusoids of a single frequency. But the theory is linear, and hence, superpositions of such sinusoids are also valid solutions. This opens a very large parameter space. For example, one could consider all heterogeneity signals that are formed by assigning a linear slope *β* to the power‐spectral density within the wavelength band associated with mantle heterogeneity (e.g., a white spectrum; Gurnis, 1988).

**Figure 10:**
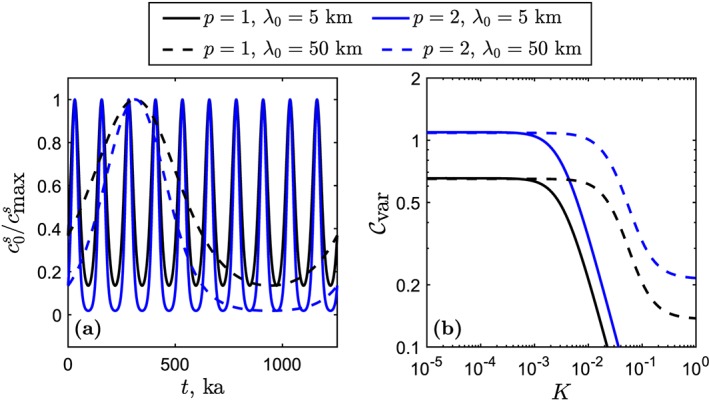
The log‐sinusoidal heterogeneity signal for p = 1,2 and λ_0_=5,50 km. (a) The heterogeneity signal as a function of time. (b) The coefficient of variation at the top of the column as a function of partition coefficient. The dry column uses W_0_=4 cm/year, $R/\Gamma_{0}=0$, and other parameters as in Figure 1.

For present purposes, we adopt a simpler approach: We choose a periodic function that can be tuned to give a suitable maximum variance. Hence, it is sufficient for comparison with the data distribution but without additional, unconstrained complexity. In particular, we propose the following log‐sinusoidal form for the source heterogeneity,

(20) $$c_{0}^{s}(t)=c_{\max}^{s}e^{p\left(\sin\Omega t-1\right)},$$

for *p* > 0, where $c_{\max}^{s}$ is the maximum concentration (which does not need to be specified). This function is plotted for two values of *p* and two values of Ω_0_=2*π* *W*_0_/*λ*_0_ in Figure 10a. It is similar in form to the Gaussian pulse‐train proposed by Liang and Liu (2018).

Since the geochemical column models developed above are based on a time‐dependence expressed by e^*i*Ω*t*^, we express the synthetic heterogeneity function (20) in terms of the coefficients of a Fourier series

(21) $$c_{0}^{s}(z=z_{0},t)={\bar{c}}_{0}^{s}+\overset{\infty }{\underset{j=1}{\sum }}(a_{j}\cos j\Omega_{0}t+b_{j}\sin j\Omega_{0}t).$$

Coefficients *a*_*j*_ and *b*_*j*_ are determined numerically.

The liquid concentrations at the column top can also be expressed as a Fourier expansion, but with different coefficients, $a_{j}^{\prime },b_{j}^{\prime }$. Because the column model is linear, the primed Fourier coefficients are related to unprimed coefficients by

(22) $$a_{j}^{\prime }+ib_{j}^{\prime }=A^{\ell }(\lambda_{0}|K,R)e^{i\Delta \theta_{j}}\times (a_{j}+ib_{j}),$$

where Δ*θ*_*j*_ is the phase‐angle difference between the column bottom and top for each mode. The primed coefficients and the column‐top mean liquid concentration are used to invert the Fourier series for the concentration time series at the top of the column.

### 4.2 Geochemical Data

We consider measurements of trace‐element concentrations in mantle‐derived basalts from three data sets that, in broad terms, represent three different timescales of magma genesis, segregation, and eruption.

The first, termed the *Iceland Single Eruption* contains olivine‐hosted melt‐inclusion data from the Haleyjabunga eruption of southern Iceland (Neave et al., 2018). Melt inclusions may capture more mantle‐derived variability in melt chemistry compared with their associated whole rock, because they are trapped before extensive crustal mixing has occurred (e.g., Maclennan, 2008a; Sobolev, 1996; Sobolev & Shimizu, 1993). Iceland's geology provides a unique constraint on magma residence time in its crust: Glacial unloading at the end of the last ice age generated enhanced melting in the shallow melting region, supplying a burst of incompatible‐element‐depleted melts (Jull & McKenzie, 1996). These melts erupted within 1,000 years of deglaciation occurring (Maclennan et al., 2002), which provides the upper bound on the source‐to‐surface magma transport and residence time beneath Iceland. This timescale is effectively instantaneous in terms of solid mantle upwelling.

The second data set, termed *Iceland Multiple Eruptions*, uses the compilation from Shorttle and Maclennan (2011) and includes whole‐rock data from Iceland's northern neovolcanic zone. These glacial and postglacial eruptions represent a medium timescale of mantle sampling of probably less than 100 kyr.

The third data set, termed *MORB Series*, comes from Cordier et al. (2010), who analyzed samples from the Central Indian Ridge, which spreads at a full rate of 42 mm/year (DeMets et al., 1990). Off‐axis samples, collected by submersible, extend their record back ∼800 kyr. They document a chemical periodicity that is symmetric across the ridge axis at a period of 150–200 ka. Multiplying by an appropriate corner‐flow upwelling speed, this periodicity would correspond to mantle heterogeneity at a wavelength of order 10 km.

**Figure 11:**
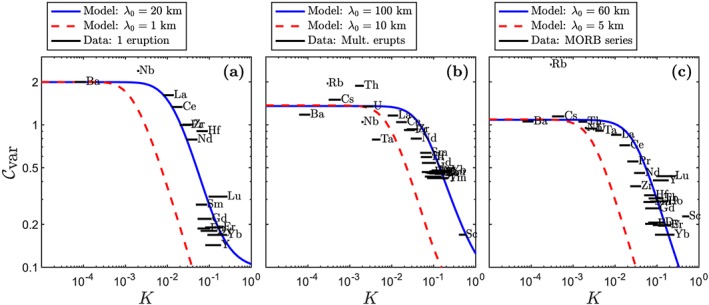
Coefficient of variation $C_{\mathrm{var}}$ for various trace elements in three different data sets. Model curves are overlayed. Solid blue curves represent numerical results fitted to data by adjusting the heterogeneity wavelength only. Dashed red curves represent numerical results with a closest timescale to geochemical data. Black lines mark $C_{\mathrm{var}}$ of geochemical data with a range of K. (a) Iceland Single Eruption (Neave et al., 2018); (b) Iceland Multiple Eruption (Shorttle & Maclennan, 2011); (c) MORB Series (Cordier et al., 2010). An upwelling rate W_0_ of 1 m/year is assumed for the Iceland models whereas a value of 2.8 cm/year is chosen for the Central Indian Ridge. Partition coefficients are from Neave et al. (2018), with the width of the bar representing the range in partition coefficient between spinel‐ and garnet‐field melting.

Data are plotted in Figure 11, with the three data sets shown separately in panels (a)–(c). For any trace element, the samples in each data set are distributed roughly according to a log‐normal distribution. The distribution for each element is summarized in terms of the coefficient of variation $C_{\mathrm{var}}$,

(30) $$C_{\mathrm{var}}=\sigma /\mu ,$$

where *σ* is the standard deviation of the concentrations and *μ* is the mean. This formula is applied to the data and the models. In Figure 11, $C_{\mathrm{var}}$ is plotted as a function of the bulk partition coefficient. For each trace element in the data, *K* is estimated using a peridotitic mineralogy. The uncertainty in *K* represents the difference between partitioning in the garnet and spinel stability fields (garnet generally gives a higher *K*).

The data in Figure 11 show an obvious trend with partition coefficient. At small *K*, the coefficient of variability is large—between one and two times the mean. There is some scatter in $C_{\mathrm{var}}$, but it generally shows a plateau for $K≲10^{-2}$; at higher values of *K*, $C_{\mathrm{var}}$ declines sharply.

Modeling results are compared with data in Figure 11. Solid blue curves represent the best‐fitting numerical results for each data set, while red dashed curves are numerical results with a wavelength that is closest to the geochemical timescale of the data. Panel (a) shows the comparison with Iceland Single Eruption; *p* = 8 in this model. The best‐fitting curve has a heterogeneity wavelength of 20 km and shows a good fit to the data, reasonably matching all elements except for niobium. However, the timescale of chemical variation at the column top that is associated with *λ*_0_=20 km is *τ* = *λ*_0_/2*W*_0_≈20 kyr (assuming an upwelling rate of 1 m/year). It is very unlikely that melt inclusions from 20 kyr of magmatic accumulation would appear in the same eruption. A more realistic period of accumulation is less than 1 kyr, represented by the red dashed curve. However, this timescale corresponds to a smaller wavelength of heterogeneity that is more attenuated than observed. This red curve could be shifted to larger admittance by assuming a smaller permeability *k*_0_ (as in Figure 7). However, to shift upward by a factor of ≳5, as required to fit the data, would mean decreasing *k*_0_=10^−12^ m^2^ by between 2 and 3 orders of magnitude. We recall that *k*_0_ is the permeability at reference porosity *ϕ*_0_=1% and that the reference speed of melt segregation is then *w*_0_=*k*_0_Δ*ρ* *g*/*ϕ*_0_ *μ*. Hence, *k*_0_=10^−14^ m^2^ corresponds to a speed of about 2 mm/year, which is inconsistent with constraints from uranium‐series disequilibrium (Stracke et al., 2006).

Figure 11b compares the Iceland Multiple Eruption data set with models using *p* = 3. The best‐fitting curve, with a wavelength of 100 km and a timescale of 100 kyr, can fit most of the data. This wavelength also corresponds to a reasonable geochemical timescale. The model curve based on a wavelength of *λ*_0_=10 km provides a poor fit to the data but has an acceptable geochemical timescale (∼10 kyr).

Data/model comparison with MORB series in panel (c) is consistent with the comparison for Icelandic basalts. The geochemical periodicity (∼175 ka) that was identified by Cordier et al. (2010) is associated with a ∼5‐km wavelength of heterogeneity (assuming an upwelling rate of 2.8 cm/year), whereas the best fitting wavelength of 60 km would have a periodicity of 2.1 Myr, longer than the timescale sampled by the entire data set (800 kyr; Cordier et al., 2010). A value of *p* = 2 is used in this case.

Curves in Figure 11 are computed with a dry column model with constant isentropic productivity. If we had instead used the wet model, the admittance at all but the smallest partition coefficients would be reduced. To compensate for this, a larger wavelength would be needed to fit the observations. This would put the model even further outside the timescale constraints associated with the data.

## 5 Discussion

In this section we discuss aspects of the results above, in comparison with observations and with other relevant constraints. We summarize the systematics of the model and highlight its deficiencies (in the narrow sense of the approximations made). We then discuss the model in the broader context of models that could plausibly explain the observations, including the other end‐member explanation of heterogeneity of melt‐transport processes. We conclude with some remarks on the path forward.

### 5.1 A Correct and Sufficient Explanation of the Observations?

Evidently, the column models (and synthetic heterogeneity) developed here can provide a good fit to the variability spectrum of trace elements in several natural settings. This is because the models and data share two key characteristics: first, a plateau in the coefficient of variability at the smallest partition coefficients and, second, a sharp drop‐off in variability with increasing partition coefficient. The model is matched to these characteristics by adjusting the *p* value of the synthetic heterogeneity, which controls the sharpness of the enriched peaks, and its fundamental wavelength *λ*_0_. The former sets the height of the plateau in $C_{\mathrm{var}}$ at small *K* whereas the latter controls the position of the drop‐off in $C_{\mathrm{var}}$ at larger *K*.

Does the goodness of fit between models and data, then, indicate that the models are a correct and sufficient explanation for the observations? Almost certainly not. The synthetic heterogeneity used here is undoubtedly oversimplified from the natural system, but we have few constraints on what it really should be. Moreover, since we consider only variability for each trace element, there are other synthetic patterns that would have worked equally well (e.g., the family with the same power spectrum but with randomized phase angles). The more significant problem is the fundamental wavelength, *λ*_0_.

The best‐fitting wavelengths in Figure 11 are relatively large, which gives rise to an important discrepancy with observations. Consider, first, the single eruption in panel (a). For a best‐fitting wavelength of 20 km and an upwelling speed of 1 m/year, the period of chemical oscillation in our column model would be 20 ka. In contrast, the melt‐extraction timescale in Iceland is probably on the order of thousands of years. The magma that was captured in the melt inclusions of the single eruption analyzed by Neave et al. (2018) was probably generated over a period similar to the melt‐transport timescale — a factor of 20 smaller than suggested by the model. A heterogeneity wavelength that is consistent with the melt‐transport timescale, *λ*_0_=1 km, gives a model outcome that is inconsistent with observations.

The MORB series from the Central Indian Ridge (Figure 11c) presents a similar issue. The best fitting wavelength corresponds to a period of just over two million years (assuming upwelling at 2.8 cm/year). But the time span of the observations, judging from the spreading rate and the off‐axis distance, is about one million years (Cordier et al., 2010). Moreover, there appear to be about five geochemical cycles within this period, rather than the half cycle that would be predicted for *λ*_0_=60 km. So again, the time period associated with the best‐fitting wavelength represents a discrepancy with observations. Taking a wavelength of 5 km to roughly match the period of the observed geochemical cycle leads to a model $C_{\mathrm{var}}$ curve that is inconsistent with the data.

The same issues apply in comparison between the model and the Iceland Multiple Eruptions series (Figure 11b), though it is less severe. The time span of the eruptions is ∼100 ka, which is the same as the period of the best‐fitting oscillation (for upwelling at 1 m/year). This means that a single cycle of heterogeneity has passed through the system during the recorded eruptions. The data, however, show no evidence for the systematic temporal variation that might be expected with this period (Shorttle & Maclennan, 2011). A heterogeneity wavelength of 10 km, also plotted in panel (b), provides a poor fit to the data. However, it gives an indication of the model sensitivity to wavelength: The curve denoting $C_{\mathrm{var}}$ shifts to smaller *K* by 1 order of magnitude, which is as predicted by our asymptotic model (equation (17)).

It is unlikely that the contribution of off‐axis melting would resolve this discrepancy. Lateral focusing of magma (e.g., Sparks & Parmentier, 1991) brings the output of off‐axis columns to the ridge axis, where it presumably mixes with the melt produced directly below the ridge. Off‐axis columns are shorter and melt to lower *F*_max_. However, at moderate distances off axis and for most incompatible elements we have *F*_max_/*K* ≫ 1, and so the admittance spectrum should be similar on‐ and off‐axis. More importantly, if the pattern of heterogeneity in the mantle is isotropic (i.e., equant heterogeneities), then we expect incoherence of phase‐angle between on‐ and off‐axis columns. Indeed, mantle heterogeneities would need to be elongate and roughly subparallel to the base of the lithosphere for their signal to sum coherently at the ridge axis. There is no reason to expect this to be the case; indeed a priori, incoherence and cancelation are the most likely scenario. This is especially true at wavelengths smaller than the maximum lateral focusing distance. Accounting for melt from off‐axis columns would thus increase the discrepancy with the observed time‐scale.

Therefore, while the good correspondence between models and observations in Figure 11 is intriguing, it cannot be interpreted as a validation of the model. The end‐member of filtration of trace‐element heterogeneity by vertical migration and aggregation of fractional melts is not a sufficient explanation for the observations. Despite this, the comparison does not exclude the possibility that such filtration contributes to observed patterns. Indeed, it may be possible to discern its effects in more elaborated models such as those discussed below.

### 5.2 Model Systematics and Limitations

We here summarize and critique the model proposed above.

Our definition of admittance means that the filtration properties of the melting column are captured by its systematics. This is best summarized by the asymptotic solution for infinite permeability (equation (17)). It shows that attenuation of amplitude for a particular mode is expected when the wavelength of that mode is small compared to the height of the transfer regime. A smaller *K* means a shorter transfer regime and hence less attenuation of heterogeneity at a given wavelength. Small amounts of reactive equilibration enhance the attenuation of heterogeneity. It is only at the highest reaction rates (e.g., for grain sizes of tens of microns) that near‐chromatographic transport occurs, preserving heterogeneity at all wavelengths.

The asymptotic solution assumes that the isentropic productivity is uniform with depth. This is a reasonable approximation for a dry melting column, but not when volatiles are present. In that case, a low‐productivity zone appears at the base of the melting region and lengthens the transfer regime. Porosity, permeability, and hence melt segregation are small in this zone compared with the silicate melting region above. Nonetheless, segregation over the longer transfer regime reduces the admittance for most *K* values. The alternative scenario to this is one where productivity is high at the base of the melting region, such as occurs for some pyroxenitic lithologies (e.g., Lambart et al., 2016). In this case, the transfer regime would be diminished in height; melt segregation would be enhanced by higher porosity, but the overall effect would be to increase admittance for most *K* values. This highlights the importance of melt productivity at the onset of melting for attenuation of source heterogeneity.

The present model is clearly an end‐member of the possible models for trace‐element variability in basalts. Below we discuss it within this broader context. However, even in the narrow confines of one‐dimensional column models, there is an assumption made above that should be questioned. We have postulated fractional melt production (*c* ^Γ^=*c* ^*s*^/*K*) while also requiring negligible reactive equilibration (R=0). However, production of incremental melts that are in equilibrium with the solid concentration requires that trace‐element mass is rapidly transferred from the interior of solid grains to their rim, which contradicts the choice of R=0. A treatment of incremental melts consistent with R=0 is *c* ^Γ^=*c* ^*s*^, where *c* ^*s*^ is the mean concentration of the solid. If the solid grains are initially uniform in concentration, then, with this combination of *c* ^Γ^ and R, they remain uniform; hence, the concentration at the rim of the grain is equal to the mean concentration. But this concentration can be far from obeying the partitioning behavior that is observed in laboratory experiments and natural lavas; it is therefore dismissed on empirical grounds.

Previous workers have proposed models that reconcile these contradictions. This class of models resolve the solid concentration as a function of radius within the interior of representative grains (e.g., Iwamori, 1993; Liang, 2003; Qin, 1992). Chemical diffusion in the radial direction allows for transport of trace elements to the rim of the grain, where they are transferred to the melt according to their concentration (and concentration gradient) at the rim. This approach should be applied to the problem of sinusoidal variation of trace elements in the source. While that is beyond the present scope, it is worth considering the timescales associated with the relevant processes: intragrain diffusion of concentration, melting from *F* = 0 to *F* = *K*, and variation of concentration by melt segregation from a heterogeneous source. These can be written:

(24a) $$\tau_{\text{difn}}∼\frac{a^{2}}{4\pi D^{s}}\approx 200\text{ka},$$

(24b) $$\tau_{\text{melt}}∼\frac{Kz_{0}}{F_{\max}W_{0}}\approx 800\text{ka},$$

(24c) $$\tau_{\text{hetr}}∼\frac{\lambda_{0}}{W_{0}}\approx 100\text{ka},\;$$

where we have used grain size *a* = 3 mm, diffusivity $D^{s}=10^{-19}$ m^2^/s, partition coefficient *K* = 0.01, column height *z*_0_=70 km, maximum degree of melting *F*_max_=0.23, upwelling speed *W*_0_=4 cm/year, and source heterogeneity wavelength *λ*_0_=5 km. The ratio *τ*_difn_/*τ*_melt_≈1/4 tells us that diffusion is moderately faster than melting. It is independent of the wavelength of heterogeneity but is sensitive to the grain size. For the assumption of *c* ^Γ^=*c* ^*s*^/*K* to be justified, we do need diffusion within the grain to be much faster than melting.

The ratio *τ*_difn_/*τ*_hetr_≈2 tells us that diffusion is commensurate with or slightly slower than fluctuations due to heterogeneity. This number depends on grain size and heterogeneity wavelength. To properly justify the assumption of R=0, the timescale of diffusion should be much greater than that of chemical variability due to heterogeneity (so *τ*_difn_/*τ*_hetr_ should be very large). From these arguments we can conclude that the model assumptions made here, while effective for simplifying the problem, cannot be justified robustly on the basis of scaling arguments.

However, geochemical observations of mean trace‐element concentrations have long been interpreted in terms of fractional melting. Therefore, this assumption is scientifically relevant. Further work is needed to develop a theory for admittance of trace‐element heterogeneity in the context of grain‐resolving models, building on the existing literature (e.g., Qin, 1992; Iwamori, 1993; Liang, 2003).

Other column models, going back at least to McKenzie (1985), have allowed for a parameterized lateral transport of magma into isolated channels with rapid transport to the surface. This approach has been further formalized in terms of a *double‐porosity* theory, with overlapping and coupled continua representing the high‐permeability channels and the low‐permeability interchannel regions separately (Liang & Parmentier, 2010). Pseudo‐two‐dimensional models by Liu and Liang (2017) apply the double‐porosity theory to isotope systems beneath a mid‐ocean ridge. Models with one porosity field that resolve the dynamics in 2‐D show that channelized transport can generate chemical variability from a homogeneous mantle (Spiegelman & Kelemen, 2003). However, Liang et al. (2011) cautioned that porosity waves associated with reactive flow can promote dispersion and mixing of chemical heterogeneities. Liang and Liu (2018) found that an isolated chemical anomaly gets extensively stretched when it is carried by magma within a channel. Indeed, channels will aggregate magmas vertically, as in the model here, but will also aggregate laterally by their suction. The present formulation could be extended to include parameterized channel flow, but lateral aggregation of diverse melts would require a two or three‐dimensional domain.

Finally, we emphasize that in natural systems, the mantle source and melt transport are almost certainly heterogeneous. These phenomena will likely be coupled through lithological heterogeneity of the source that, by creating productivity heterogeneity, may cause lateral variability in melt transport rates and structure (Katz & Weatherley, 2012; Kogiso et al., 2004; Lundstrom et al., 2000; Weatherley & Katz, 2012). This potentially creates a complex interaction between basalt chemistry and its transport through the mantle. If basalt chemistry is evaluated with this coupled interaction in mind, then its interpretation in terms of quantitative estimates of source components becomes more challenging (e.g., Shorttle et al., 2014). However, at a global scale, some geochemical evidence suggests that major element heterogeneity of the mantle is relatively inconsequential compared to thermal heterogeneity (e.g., Gale et al., 2014). Given our limited ability to resolve the lithologies involved in melting and characterize their melting behaviors, direct study of the chemical transport associated with a heterogeneous mantle is not yet tractable.

### 5.3 Causes of Geochemical Variability in Basalts

The present work presents an end‐member case that quantifies the homogenizing potential of vertical melt aggregation. Addition of further complexity in terms of parameterized channel flow would not serve this purpose and hence has been avoided. By comparison of our limited model with observations, we falsify the hypothesis that source heterogeneity alone (i.e., in the absence of temporal or spatial heterogeneity of melt transport) can account for variability in melts delivered from the mantle.

Incremental fractional melts of a homogeneous mantle span a very large range of concentrations from highly enriched (deepest, incipient melts) to highly depleted (shallowest melts). Aggregation with vertical transport averages this variability. Channels that transport deep melts to the surface with limited aggregation of shallower melts are thus an appealing hypothesis for the observed variability. Models of channelized flow (e.g., Aharonov et al., 1995; Spiegelman et al., 2001) were shown by Jull et al. (2002) and Spiegelman and Kelemen (2003) to deliver very large trace‐element variability to the crust. The present results lend support to this hypothesis by demonstrating the shortcomings of a transport model without channelization.

Channels emerge because of a positive feedback between vertical flux, reactive melting, and porosity (permeability) growth. The magma in channels is underpressured due to their high permeability and vertical extent. This underpressure draws in melts laterally (and also drives compaction; see Rees Jones & Katz, 2018). Reactive melting persists in channels until pyroxene has been exhausted from the residue. It remains unclear whether, in the absence of in situ melting, a lateral influx of melt is sufficient to maintain open channels at steady state (Liang et al., 2010). Regardless, it is evident that aggregation of melts occurs even in a channel. The theory presented above should also be relevant for understanding the consequences of that aggregation.

Moreover, the depth to which channels penetrate remains poorly constrained (though see Jull et al., 2002). It may be impossible for channels to reach the base of the melting regime, where the segregation melt flux is small. If channels penetrate to an intermediate depth within the melting region, there could be homogeneous melt transport below that depth. Trace elements with sufficiently small *K* would then have a transfer regime that is entirely deeper than the onset of channels. For those trace elements, the model developed here would be useful in predicting how source heterogeneity is admitted (or attenuated) in deep melts before they enter channels.

A key factor that complicates these considerations is that the mantle is heterogeneous in major elements as well as trace elements. Indeed, source variations of trace and major elements may derive from the same process and therefore have tight spatial correlation (e.g., Hirschmann & Stolper, 1996; Langmuir et al., 1980; Shorttle & Maclennan, 2011). Major element variability affects the fusibility of the mantle, and hence the distribution of productivity with depth. Melting of fertile domains may be fueled by heat from surrounding, refractory regions (Katz & Rudge, 2011). Melt derived from fertile domains could promote channelization (e.g., Katz & Weatherley, 2012; Lundstrom et al., 2000; Weatherley & Katz, 2012) or magmatic waves. Jordan et al. (2018) has shown that solitary magmatic waves may be able to trap and transport geochemical signals in isolation from surrounding melts. Hence, it seems likely that a comprehensive explanation for geochemical variations in erupted basalts should account for both source and transport heterogeneity, and their interaction. This remains a major challenge.

Clarifying the behavior of end‐member models of geochemical variability is a useful step toward this goal. Here we have emphasized the variability of trace‐element concentrations, for which there are many measurements. A consideration of stable and radiogenic isotopes, while adding some complexity to the problem, may ultimately be necessary to disentangle the physical processes involved in melt extraction from a heterogeneous mantle. Future models should incorporate such tracers and should explore the space of models that incorporate heterogeneity of both the mantle source and of the melt transport process.

## Figures and Tables

**Figure D1:**
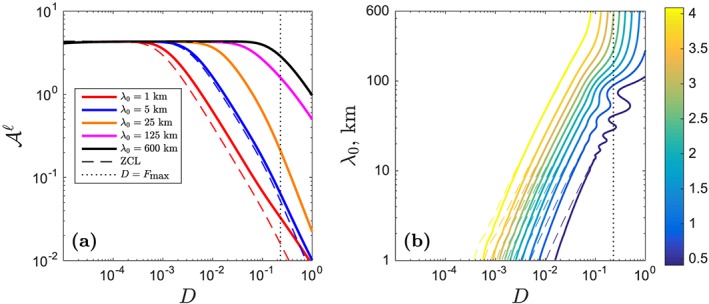
Admittance $A^{\ell }$ calculated for a dry melting column with a numerical solution that considers both the compaction stress and the Darcy drag. All other parameters are identical to Figure 2. (a) $A^{\ell }$ as a function of partition coefficient K for various wavelengths of heterogeneity, as in legend. (b) Contours of constant $A^{\ell }$ as a function of K and input heterogeneity wavelength λ_0_. Other parameters are the same as in Figure 1.

## Appendix A Melting Column Models

### A.1

A melting column is typically defined in the context of a mid‐ocean ridge or mantle plume, where melting occurs as a consequence of isentropic decompression of the upwelling solid mantle. The column is a one‐dimensional domain, aligned with gravity, in which we solve the steady, Boussinesq, two‐phase equations (e.g., Ribe, 1985). Mass conservation for the liquid and solid phases is expressed as

(A1a) $$\;\;\;\;\frac{d}{dz}\left(\mathrm{\phi w}\right)=\Gamma /\rho ,$$

(A1b) $$\frac{d}{dz}\left[(1-\phi )W\right]=-\Gamma /\rho .$$

Defining the degree of melting as

(A2) $$F(z)\equiv \frac{\int_{z_{0}}^{z}\Gamma (z^{\prime })\;dz^{\prime }}{\rho W_{0}},$$

the mass conservation equations can be integrated to give

(A3a) $$(1-\phi )W=W_{0}(1-F),$$

(A3b) $$\mathrm{\phi w}=W_{0}F.$$

The momentum conservation equation for the two‐phase aggregate is derived by combining the Darcy‐like balance for the liquid phase with the Stokes‐like balance for the solid phase (McKenzie, 1984) to give

(A4) $$\phi (w-W)=k_{\phi }(1-\phi )\Delta \mathrm{\rho g}/\mu ,$$

where Δ*ρ* is the density difference between solid and liquid, *g* is gravitational acceleration, and *μ* is the magma viscosity; the *z*‐direction is chosen to be positive upwards (opposite to gravity). The permeability is given by

(A5) $$k_{\phi }\equiv k_{0}{\left(\phi /\phi_{0}\right)}^{n},$$

where *k*_0_ is the permeability when the porosity is equal to the reference porosity *ϕ*_0_ and *n* = 2 is a constant that we fix according to empirical and theoretical results for small porosity (e.g., Miller et al., 2014; Rudge, 2018; von Bargen & Waff, 1986). Equation (A4) is derived by making the zero‐compaction‐length approximation in which compaction stresses are neglected relative to Darcy drag (Ribe, 1985; Spiegelman, 1993).

Combining the integrated mass conservation equations (A3) with (A4) gives us the implicit solution written in equation (10) for *ϕ*(*z*),*w*(*z*), and *W*(*z*). A melting model then determines Γ and closes the equations. In Appendix B we prescribe Γ by imposing a constant isentropic productivity; in Appendix C we develop a melting model for Γ that includes the effect of volatile elements.

## Appendix B A Simple Column With Analytical Constraints on Admittance

Here we assume that the melting rate is driven by bulk decompression. In particular, we take Γ=*ρ* *W*_0_Π, where Π is a constant, uniform, isentropic productivity of upwelling. Then, *F*=Π(*z* − *z*_0_)≡Π*Z*. Here we have defined *Z* as the dimensional height above the bottom of the column.

An explicit, analytical solution to equation (10) can be obtained for *n* = 2 or 3. The former is more appropriate at very small porosity (Rudge, 2018). In Figure B1, we plot the *n* = 2 solution to the system of equation (10).

With this column model and neglecting exchange reactions ( X=0), equation (7) for the mean concentration become

(B1a) $$\;\frac{d{\bar{c}}^{s}}{dF}=-\left(1/K-1\right)\frac{{\bar{c}}^{s}}{1-F},$$

(B1b) $$\frac{d{\bar{c}}^{\ell }}{dF}=-\frac{{\bar{c}}^{\ell }-{\bar{c}}^{s}/K}{F}.\;\;$$

Application of the time‐independent part of the boundary condition (1) yields the solution

(B2a) $${\bar{c}}^{s}(F)={\bar{c}}_{0}^{s}{\left(1-F\right)}^{1/K-1},$$

(B2b) $${\bar{c}}^{\ell }(F)={\bar{c}}_{0}^{s}\frac{1-{\left(1-F\right)}^{1/K}}{F}.$$

These are the equations of aggregated fractional melting (Shaw, 2006).

The equations for the fluctuation amplitudes follow from (8) using mass conservation equations (A3),

(B3a) $$\;\;\frac{d{\overset{˘}{c}}^{s}}{dZ}=-\frac{\Pi {\overset{˘}{c}}^{s}}{1-\Pi Z}\left(\frac{1}{K}-1\right)-i\frac{\Omega {\overset{˘}{c}}^{s}}{W(Z)},$$

(B3b) $$\frac{d{\overset{˘}{c}}^{\ell }}{dZ}=-\frac{{\overset{˘}{c}}^{\ell }-{\overset{˘}{c}}^{s}/K}{Z}-i\frac{\Omega {\overset{˘}{c}}^{\ell }}{w(Z)}.$$

Using the fluctuating part of the boundary condition (1) and *F*=Π*Z* gives the solution

(B4a) $${\overset{˘}{c}}^{s}(F)={\overset{˘}{c}}_{0}^{s}e^{-i\Omega t^{s}(F)}{\left(1-F\right)}^{1/K-1},\;\;\;$$

(B4b) $$\;\;{\overset{˘}{c}}^{\ell }(F)={\overset{˘}{c}}_{0}^{s}F^{-1}e^{-i\Omega t^{\ell }(F)}\int_{0}^{F}e^{-i\Omega \Delta t(F^{\prime })}\frac{1}{D}{\left(1-F^{\prime }\right)}^{1/K-1}dF^{\prime }.$$

Here we have introduced

(B5) $$t^{s}(F)\equiv \int_{0}^{F}\frac{1}{W(F^{\prime })}\frac{dF^{\prime }}{\Pi },\;t^{\ell }(F)\equiv \int_{0}^{F}\frac{1}{w(F^{\prime })}\frac{dF^{\prime }}{\Pi },$$

as the time for a parcel of solid or liquid, respectively, to move from the bottom of the melting column (*F* = 0) to the height where the degree of melting is *F*. We also defined Δ*t* ≡ *t* ^*s*^−*t* ^*ℓ*^. These definitions allow us to avoid specifying upwelling rates, for the moment.

Using the solution (B4), we can evaluate the admittances defined in equation (9). For the solid phase,

(B6) $$A^{s}(F)={\left(1-F\right)}^{1/K-1}.$$

From this expression and the solution B2a, it is evident that at any height in the column, $A^{s}$ is equal to ${\bar{c}}^{s}(Z)/{\bar{c}}_{0}^{s}$. This means that for the solid phase, the decay of concentration fluctuations with height in the melting column is identical to the decay of the mean concentration.

For the liquid phase,

(B7) $$A^{\ell }=\frac{\left|{\overset{˘}{c}}^{\ell }(F)\right|}{\left|{\overset{˘}{c}}_{0}^{s}\right|}=\frac{1}{F}\left|\int_{0}^{F}e^{-i\Omega \Delta t(F^{\prime })}\frac{1}{K}{\left(1-F^{\prime }\right)}^{1/K-1}dF^{\prime }\right|.$$

This equation cannot be evaluated analytically without approximations. It can, however, be bounded according to

(B8) $$A^{\ell }\leq \frac{1}{\mathrm{KZ}}\int_{0}^{Z}\left|{\left(1-\Pi Z\right)}^{\left[1/K-1+i\Omega /(\prod W_{0})\right]}e^{i\Omega \int_{0}^{Z}(1/w)dZ}\right|dZ=\frac{1-{\left(1-\Pi Z\right)}^{1/K}}{\Pi Z},$$

where we have used *F*=Π*Z* and the integral inequality $\left|\int f\;dz\right|\leq \int \left|f\right|\left|dz\right|$. Comparing (B8) with the solution B2b for the mean liquid concentration,

(B9) $$A^{\ell }(F)\leq {\bar{c}}^{\ell }(F)/{\bar{c}}_{0}^{s}.$$

Therefore, we conclude that for the liquid phase, the decay of concentration fluctuations with height in the melting column is at least as rapid as the decay of the mean liquid concentration.

We make further progress by introducing assumptions that simplify the integrand of (B7). We first consider the quantity Δ*t*, which represents the time difference for transport of the solid and liquid phases between *Z* = 0 and *Z* = *F*/Π. It is expanded as

(B10) $$\Delta t(F)=\int_{0}^{F}\left(\frac{1}{W(F^{\prime })}-\frac{1}{w(F^{\prime })}\right)\frac{dF^{\prime }}{\Pi }\equiv G(F),$$

where G(F) is an unknown, nonlinear function. When $k_{0}\to \infty$, the permeability is infinite and 1/*W* ≫ 1/*w* at all *F* > 0. In this limiting case *t* ^*ℓ*^∼0, and we find, using A3a, that $G(F)∼-\ln(1-F)/(W_{0}\Pi )$. This can be further simplified when *F* ≪ 1 to give

(B11) $$G(F)∼\frac{F}{W_{0}\Pi }\equiv G_{0}F.$$

This result simply means that the travel time difference at any height *Z* is approximated by the travel time of the solid at the background upwelling speed: Δ*t* ∼ (*z* − *z*_0_)/*W*_0_.

We also introduce the approximation ${\left(1-F\right)}^{1/K-1}\approx e^{-F/K}$, which requires the additional assumption that *K* is much smaller than unity. For the purposes of this manuscript, it is adequate that K≲0.1 for F≲0.2.

Using the these approximations to rewrite equation (B7) gives

(B12) $$A^{\ell }∼\frac{1}{F}\left|\int_{0}^{F}\frac{e^{-(i\Omega G_{0}+1/K)F^{\prime }}}{K}dF^{\prime }\right|.$$

This integral can be evaluated to give

(B13) $$A^{\ell }∼\frac{1}{F}\frac{\sqrt{{\left(1-e^{-F/K}\right)}^{2}+4e^{-F/K}\sin^{2}\left(FG_{0}\Omega /2\right)}}{\sqrt{1+{\left(KG_{0}\Omega \right)}^{2}}}.$$

Recall that this asymptotic result is strictly valid for *K*,*F* ≪ 1 and $k_{0}\to \infty$. For highly incompatible elements with *K* ≪ *F*, we can simplify further and obtain

(B14) $$A^{\ell }∼\frac{1}{F\sqrt{1+{\left(KG_{0}\Omega \right)}^{2}}}\;\left(\text{for}\;K\ll F\right).$$

The simple approximations (B13) and (B14) capture the structure of the admittance well when R=0 and the melt productivity is constant. They are plotted in Figures 1 and 2 and discussed in section 3.1 of the main text.

## Appendix C A Melting Column With Volatiles

To incorporate the effect of volatile elements on the steady‐state porosity and velocity profiles in the column, we append a simple thermochemical model to equations (A1) and (A4), following the approach of Rees Jones et al. (2018). This uses a steady‐state conservation of energy, written in terms of temperature *T* as

(C1) $$\rho c_{P}W_{0}\frac{dT}{dz}=-(L\Gamma +\rho \alpha W_{0}\mathrm{gT}),$$

where *c*_*P*_ is specific heat capacity, *L* is the latent heat of fusion (J/kg), and *α* is the coefficient of thermal expansion. This equation states that the advection of sensible heat by bulk upwelling of rock and magma is balanced by conversion to latent heat through melting, and conversion to work through volume expansion.

We assume that the mantle is composed of two components, *rock* and *volatiles*. Volatile concentration is denoted by a *C* (capitalized) to distinguish it from a trace‐element concentration. The solidus is the relationship between temperature, pressure, and the volatile concentration of the solid when both solid and melt are present. We assume a simple form in which this relationship is linearized about the conditions at the bottom of the column,

(C2) $$T=T_{0}^{S}-\gamma^{-1}\mathrm{\rho g}(z-z_{0})-M(C^{s}-C_{0}^{s}),$$

where *γ* is the Clausius‐Clapeyron slope and *M* is the slope of the solidus with volatile concentration; both are taken as constants. We assume a lithostatic pressure gradient. The liquidus curve is defined by the assumption of a constant ratio, $K_{w}\equiv \left[C^{s}/C^{\ell }\right]$, between the volatile concentration in the solid and the liquid. Hence, the equilibrium compositional difference between phases is Δ*C* = *C* ^*s*^(1 − 1/*K*_*w*_). Using the lever rule referenced to the initial concentration, we can define the degree of melting of the solid phase as $F\equiv \left(C^{s}-C_{0}^{s}\right)/\Delta C$. Then, combining this with equation (C2) and the partitioning relation for Δ*C*, we can express *F* as a function of temperature,

(C3) $$F=\frac{T-T_{0}^{S}+\gamma^{-1}\mathrm{\rho gz}}{\left(1-1/K_{w})(T-T_{0}^{S}+\gamma^{-1}\mathrm{\rho gz}+MC_{0}^{s}\right)}.$$

Using (C3), the melting rate Γ can be written as

(C4) $$\Gamma =\rho W_{0}\frac{dF}{dz}=\rho W_{0}\frac{\mathrm{\partial F}}{\mathrm{\partial T}}\left(\frac{dT}{dz}+\gamma^{-1}\mathrm{\rho g}\right).$$

Substituting this into the conservation of energy equation (C1) gives

(C5) $$\left(1+\frac{L}{c_{P}}\frac{\mathrm{\partial F}}{\mathrm{\partial T}}\right)\frac{dT}{dz}+\frac{\mathrm{\alpha g}}{c_{P}}T+\frac{\mathrm{\rho g}\gamma^{-1}L}{c_{P}}\frac{\mathrm{\partial F}}{\mathrm{\partial T}}=0.$$

Since *∂* *F*/*∂* *T* is a function of *T*, this equation is nonlinear; we integrate it numerically to find *T*(*z*), which is then used in (C3) to find *F*(*z*). Mass and momentum conservation are then used to obtain *ϕ*,*w*, and *W*. Two example solutions with different values of *k*_0_ are shown in Figure B1.

Properties of the admittance of trace‐element variations are not available by analytical methods for this melting column. We obtain results by numerical methods in section 3.2 of the main text.

## Appendix D Noncompacting Boundary Layer

All the above melting column solutions are based on the zero‐compaction‐length (ZCL) approximation, which neglects gradients in the compaction pressure. These gradients are important only in a narrow boundary layer at the bottom of the melting column (e.g., Ribe, 1985; Šrámek et al., 2007). However, it is precisely at the bottom of the melting column (in the transfer regime) where attenuation of heterogeneity occurs. Therefore, it is important to consider whether the ZCL approximation makes a qualitative difference to the results and conclusions of this study.

Within the narrow boundary layer near the onset of melting, melt buoyancy is balanced by a gradient in the compaction pressure; there is little compaction, and hence, *w* ∼ *W*_0_ and *ϕ* ∼ *F* (in the rest of the column, buoyancy is balanced by Darcy drag). Therefore, the ZCL approximation predicts liquid segregation that is too rapid, compared to the full solution, in the boundary layer. We have shown that the key factor controlling admittance is the accumulated phase difference in the transfer regime. Hence, we need to evaluate the importance of the noncompacting boundary layer (NCBL) within the transfer regime.

When the height of transfer regime is much larger than the height of the noncompacting boundary layer, for example, for a mildly incompatible trace element, the inaccuracy of the ZCL assumption is clearly negligible. For small enough *K*, the height of transfer regime will become comparable height to the NCBL. In this case, the phase difference would be reduced within the transfer regime, which would diminish the attenuation. But if the wavelength of heterogeneity is much larger than both the NCBL and the transfer regime height, then attenuation is minimal anyway. These two cases cover all combinations of *K* and *λ*_0_ considered here, and hence the ZCL approximation does not qualitatively affect our results.

To demonstrate that the quantitative effect is small, we have computed numerical solutions of melt segregation that do not neglect gradients in compaction stress (in the dry case only). These results are used in the trace element model to compute the admittance. Figure D1 shows the change in admittance from relaxing the ZCL assumption, for all other parameters held constant.
